# Biogenic synthesis of palladium nanoparticles and their applications as catalyst and antimicrobial agent

**DOI:** 10.1371/journal.pone.0184936

**Published:** 2017-09-28

**Authors:** Munmi Hazarika, Debajit Borah, Popymita Bora, Ana R. Silva, Pankaj Das

**Affiliations:** 1 Department of Chemistry, Dibrugarh University, Dibrugarh, India; 2 Centre for Biotechnology and Bioinformatics, Dibrugarh University,Dibrugarh, India; 3 CICECO-Aveiro Institute of Materials, University of Aveiro, Aveiro, Portugal; Institute of Materials Science, GERMANY

## Abstract

This paper describes a simple *in-situ* process of synthesizing highly dispersed palladium nanoparticles (PdNPs) using aqueous leaf extract of *Garciniapedunculata*Roxb as bio-reductant and starch (0.3%) as bio-stabilizer. The PdNPs are characterized by techniques like FTIR, TEM, SEM-EDX, XRD and XPS analysis. It is worthnoting thatwhen the synthesis of nanoparticles was carried out in absence of starch, agglomeration of particles has been noticed.The starch-assisted PdNPs showed excellent aqueous-phase catalytic activities for three important reactions: the Suzuki-Miyaura cross-coupling reactions of aryl halides (aryl bromides and iodides) with arylboronic acids; selective oxidations of alcohols to corresponding carbonyl compounds; and reduction of toxic Cr(VI) to nontoxic Cr(III). Our catalyst could be reused up to four cycles without much compromising with its activity. Furthermore, the material also demonstrated excellent antimicrobial and anti-biofilm activities against a novel multidrug resistant clinical bacterial isolate *Cronobactersakazakii* strain AMD04. The minimum inhibitory concentration (MIC) and minimum bactericidal concentration (MBC) of PdNPswere found to be 0.06 and 0.12 mM respectively.

## Introduction

Over the past few decades, delving into the synthesis of metalnanoparticles*viz*. gold, silver, Zinc, platinum, palladium, *etc*.have fetched considerable attention in the fields of material and biological sciences [1–8]. Amid other metal NPs, Pd is gaining special mention because of its profound applications as catalyst in large number of organic transformations which includevarious types of carbon-carbon cross-coupling [9,10], oxidation [11,12]and reduction reactions. [13,14]. Traditionally, Pd NPs are synthesized *via* various physical or chemical methods with the aid of toxic and hazardous reducing and stabilizing agents, however, over the years, in the quest of ‘going green’, there has been a paradigm shift towards bio-inspired strategies for the synthesis of metal NPs [15–17]. The biocompatibility and environmentally benign properties attributes to these biological techniques to supersede the conventional physical and wet-chemical methods. The field of biological synthesis of Pd NPs encompasses the use of plant extracts, microorganisms, marine organisms, *etc*. as green-reductants. Amongst those methods, the tapping of bio-resources, particularly the plant extracts for the synthesis of the NPs seems promising owing to their ready availability, rapid process, better cost-effectiveness, and the ability to use in large-scale biosynthesis [18]. Hence, plant mediated syntheses of PdNPs, has gained many recent attentions[19,20]. Examples include use of peel-extract of banana [21], leaf-extracts of *Pulicaria glutinosa*[16],*Perilla frutescens*[17],*Euphorbia granulate*[22],*Ocimum Sanctum*[23], *Origanum vulgare* [24],*etc*. and some of the systems have been successfully applied as catalysts for organic reactions like Suzuki-Miyaura reactions[16,21,25–28], oxidation of alcohols [24], hydrodechlorination of p-chlorophenol [29], *etc*. Unfortunately, baring a couple of examples [22,25,28], majority of these reported plant-based catalysts often suffer from limitations like high reaction temperatures (up to 120°C), high catalyst loadings (up to 12 mol%), limited substrate scopes and / or use of undesirable organic solvents as reaction, etc. Moreover, the reported plant-based Pd NPs mostly tested for a single catalytic system, while their multifunctional potentiality remains largely under-explored. Thus, the appropriate selection of plant extract which can bestow multifunctional roles *viz*., bio-reduction of Pd salts, aid in multiple catalytic reactions and afford biological activities is highly desirable. In this context, we would like to exploit a bio-resource namely *Garcinia pedunculata* Roxb aqueous leaf extract for the synthesis of Pd NPs. This leaf extract has been traditionally known to possess multiple medical uses, validated by ethno-pharmaceutical research [30]. Literature reveals the presence of phytochemical constituents like polyphenol, flavonoid, hydroxycitric acid in the leaf extract, which functions as active components for bio-reduction [31]. To the best of our knowledge, the potentiality of this plant has not been explored for synthesizing and functionalizing metal NPs till date. Herein, along with the synthesis of PdNPs, we have also explored the versatility of these NPs as multifunctional catalyst for Suzuki-Miyaura cross-coupling reactions of aryl halides, alcohol oxidation and Cr(VI) to Cr(III) reduction reactions using water as a reaction media. The antibacterial activity of the Pd NPs was also was investigated against a novel multidrug-resistant clinical bacterial isolate *Cronobacter sakazakii* strain AMD04. It is worth mentioning that the novelty of this reported synthesis of Pd NPs lies in appropriate selection of the bio-resource, which not only aids in the synthesis, but unlike other biogenic process plays a crucial role in bestowing multiple catalytic and antibacterial properties. Of note, the multifunctional catalytic aspects of the synthesized PdNPs viz., C-C cross-coupling, oxidation and reduction reactions constitute an added asset.

## Experimental materials and methods

Fresh green leaves of *G*. *pedunculata* Roxb (ESI: S1 Fig) were collected from Dergaon area (26.6969° N, 93.9853° E) of Golaghat District, Assam, India. Palladium acetate [Pd(OAc)_2_] was purchased from Merck India Pvt. Ltd. All the bacteriological media used in the present study were procured from HiMedia Laboratories Pvt. Ltd. The other common laboratory chemicals are of analytical grade and were purchased from different Indian firms. FTIR spectra (400–4000 cm^-1^) were recorded in KBr using Shimadzu (Prestige-21) spectrophotometer. The X-ray diffraction (XRD) study was performed in a Rigaku X-ray diffractometer (model: ULTIMA IV, Rigaku, Japan) with a Cu Kα X-ray source (λ = 1.54056 Å) at a generator voltage of 40 kV and a generator current of 40 mA. The Transmission electron microscopy (TEM) and high were carried out on a JEOL JEM-2011 electron microscope operated at an accelerating voltage of 200 kV. X-ray photoelectron spectroscopy (XPS) study was performed at ‘‘*Centro de Materiais da Universidade do Porto*” (Portugal), using a Kratos Axis Ultra HSA spectrometer with a non-monochromatized Mg Ka radiation (1253.6 eV). The scanning electron microscope (SEM) spectra were recorded using JEOL Model JSM - 6390LV and energy dispersive X-ray (EDX) peaks were recorded in JEOL Model JED– 2300. Bacterial growth was measured with the help of UV-VIS spectrophotometer (Shimadzu UV-1800, Japan).HPLC analysiswas done with Agilent HPLC System (Infinity 1200) equipped with Multi-Wavelength Detector. Thermogravimetric analysis (TGA) was performed using Perkin Elmer STA-8000 at the heating rate of 10°C per min under nitrogen atmosphere. Dynamic Light Scattering (DLS) study was done by using Zetasizer analyser (Model: Nano ZS, Malvern, UK) instrument.

### Preparation of plant leaf extract

100 gm fresh leaves of *G*. *pedunculata*Roxbwere chopped nicely and then added slowly in 500 mL deionized water with occasional shaking. The aqueous mixture was boiled for 30 min with continuous stirring and then allowed to cool to room temperature. The leaf extract was then filtered using Whatman no.1 filter paper and the filtrate was stored at 4°C for further use.

### Synthesis of palladium nanoparticles

500 ml aqueous leaf extract of the plant *G*. *pedunculata*Roxb was mixed with 1000 ml of palladium acetate (1mM) solution containing 0.3% of starch. The reaction mixture was allowed to stir continuously for overnight and then autoclaved at 121°C for 15 min. The color of the solution changed from orange to dark brown, consistent with the formation of PdNPs. The NPs were then separated from the aqueous suspension by centrifugation at low temperature at 30,000 *g* for 30 min and then washed twice by deionized water and finally dried in an oven at 80^o^ C.

### General procedure for the Suzuki-Miyaura reaction

Forthe Suzuki-Miyaura reaction, a 50 mL round-bottomed flask was charged with aryl halide (0.5 mmol), arylboronic acid (0.65 mmol), K_2_CO_3_ (1 mmol), catalyst (0.002 g; 0.0005 mmol of Pd) and water (4 mL) and stirred at appropriate temperature. The progress of the reaction was monitored by thin layer chromatography using aluminum coated TLC plates (Merck) under UV light. At the end of reaction, the mixture was cooled down to room temperature and the product diluted with water (10 mL) and extracted with ether (3 x 15 mL). The combined extract was washed with brine (3 x 15 mL) and dried over Na_2_SO_4_. After evaporation of the solvent under reduced pressure, the residue was subjected to column chromatography with ethyl acetate/hexane (1:9) as eluent to get the desired product.

### Reusability experiment

A 100 mL round-bottomed flask was charged with *p*-bromotoluene (2.5.mmol), phenylboronic (3.25 mmol), K_2_CO_3_ (7.5 mol), H_2_O (20 mL), catalyst (10 mg; 0.0025 mmol) and stirred at 50°C for 3 h. After the reaction was over, the reaction mixture was cooled to room temperature and then 20 mL of EtOH was added and stirred for 10 min. The catalyst was then separated by centrifugation (14,000 rpm for 15 min), washed thoroughly with EtOH-H_2_O (1:1) followed by ethyl acetate, dried under vacuum and then used for subsequent runs.

### General procedure for alcohol oxidation reaction

A 50ml round bottomed flask was charged with alcohol(1.2mmol),TBHP(2.4mmol),K_2_CO_3_(0.0075 mmol), catalyst (3mg, 0.0007 mmol) and H_2_O (10mL). The reaction was carried out at 80°C with continuous stirring and monitored by using TLC.On completion of the reaction, the mixture was extracted with ether and dried over Na_2_SO_4_.The solvent was evaporated under reduced pressure to obtain the concentrated organic product which was extracted with ethyl acetate and analyzed by gas chromatography (Agilent 7820A) with mass detector (Agilent 5975 series).

### Procedure for Cr (VI) reduction

Formic acid (0.3 ml) was added to 50ml of aqueous K_2_Cr_2_O_7_(0.001M) to prepare an acidified solution of Cr(VI). To this solution, 10ml of aqueous sodium acetate trihydrate(0.5M) was added dropwise to maintain a pH of 2.The reaction mixture was stirredat room temperature in presence of 3mg(0.0007 mmol) of the PdNP catalyst and then monitored with UV-vis spectrophotometer.

### Isolation, screening and identification of novel MDR isolates

MDR bacterial isolates were screened from clinical samples collected from Assam Medical College Hospital, Dibrugarh by observing their resistance patterns against at least four antibioticsout of the following: Metropenem (10mcg), Polymixin B (10mcg), Metronidazole (5mcg), Novobiocin (30mcg), Cefixime (5mcg), Ciprofloxacin (5mcg), Tetracycline (30mcg), Gentamycin (30mcg), Amikacin (30mcg), Rifampicin (5mcg), Erythromycin (15mcg), Ampicillin (10mcg), and Imipenem (10mcg). The isolate showing resistanceagainst maximum number of antibiotics was then identified by 16S rDNA sequencing technique using 704F forward primer (5’-GTAGCGGTGAAATGCGTAGA-3’) and 907R reverse primer (5’-CCGTCAATTCMTTTGAGTTT-3’).

### Evaluation of antimicrobial activity

Antimicrobial activity of Pd NPs was evaluated by agar plate well diffusion method by loading 10 μL of the plant extract, 1 mMPd(OAc)_2_ solution, and aqueous suspension of PdNPs in respective wells. The plates were then incubated overnight at 37°C and zone of inhibition was observed around the wells. The antimicrobial activity in terms of minimum inhibitory concentration (MIC) and minimum bactericidal concentration (MBC) of Pd NPs was examined by the standard broth dilution method[32]. The lowest concentration of Pd NPs causing significant decline in the bacterial growth as compared to that of control was considered as the MIC, while the minimum concentration of PdNPs which completely inhibits the bacterial growth was considered as MBC. Bacterial growth was measured as an increase in the absorbance of the 600 nm peak determined by a spectrophotometer at an interval of 1 h. Sterile test tubes, each containing 1 mL of LB (Luria Bertani) broth were inoculated with 100μL of freshly prepared bacterial suspension in order to maintain initial bacterial concentration (10^3^−10^4^ CFU/mL). 1 mM of PdNPs solution was prepared and diluted to 0.02, 0.04, 0.06, 0.08, 0.1, 0.12 mM in 1mL culture of above mentioned *Cronobactersakazakii*strain AMD04 and then incubated in an orbital shaker at 200 rpm and 37°C (Sartorius StedimCertomat BS-1 shaker incubator, Germany Ltd.). The control was devoid of NPs containing inoculums and LB broth.

### Evaluation of anti biofilm activity

Inhibition of biofilm formation in presence of PdNPs was determined and quantified by crystal violet staining method [33]. Overnight culture of novel MDR clinical isolate *Cronobacter sakazakii* strain AMD04 was diluted (1:100) and inoculated into a micro titre plate containing increasing concentrations of PdNPs in dilute (1:100) Luria Bertani (LB) Broth. After 24 h of incubation, the micro titre plate was washed with phosphate buffer saline (PBS) to remove the unbound cells and left to air dry. The plate was stained with 0.1% crystal violet for 10–15 minutesand then washed thoroughly in phosphate buffer saline (PBS) followed by drying at 60°C. Crystal violet was extracted using 30% acetic acid for 10–15 min and absorbance was recorded at 595nm.

### Statistical analysis

All the biological experiments related to antimicrobial study were carried out in triplicate and results are expressed in mean ± S.D. Significance of the results were checked by performing student’s *t-*test (p<0.05) using online statistical tool graphpad (https://www.graphpad.com/).

## Results and discussion

### Synthesis and characterization of palladium nanoparticles

The PdNPs are synthesized by a simple *in-situ* method treating a solution of [Pd(OAc)_2_] with aqueous leaf extract of the plant *G*. *pedunculata*Roxb in presence of 0.3 mol% of starch. Since, we have not used any external reducing agent, it is logical to assume that the phytochemical constituents performed the reduction Pd^+2^→Pd^0^. In order to investigate the phyto-constituents responsible for this reduction, we have performed HPLC analysis of the leaf extract and gallic acid was found to be the major constituent present in the bio-extract. The presence of gallic acid was further confirmed by HRMS analysis which shows a molecular ion peak [M-2H]^+^at*m/e* 168 [ESI:S2 Fig].It is worthy to note that the reducing potential of gallic acidfor synthesizing metal NPs has been reported in literature [8,34]. Hence, we believe that in our case also the gallic acid has been acted as bio-reducing agent, although the possibility of other minor constituents cannot be ruled out.

**Fig 1:**
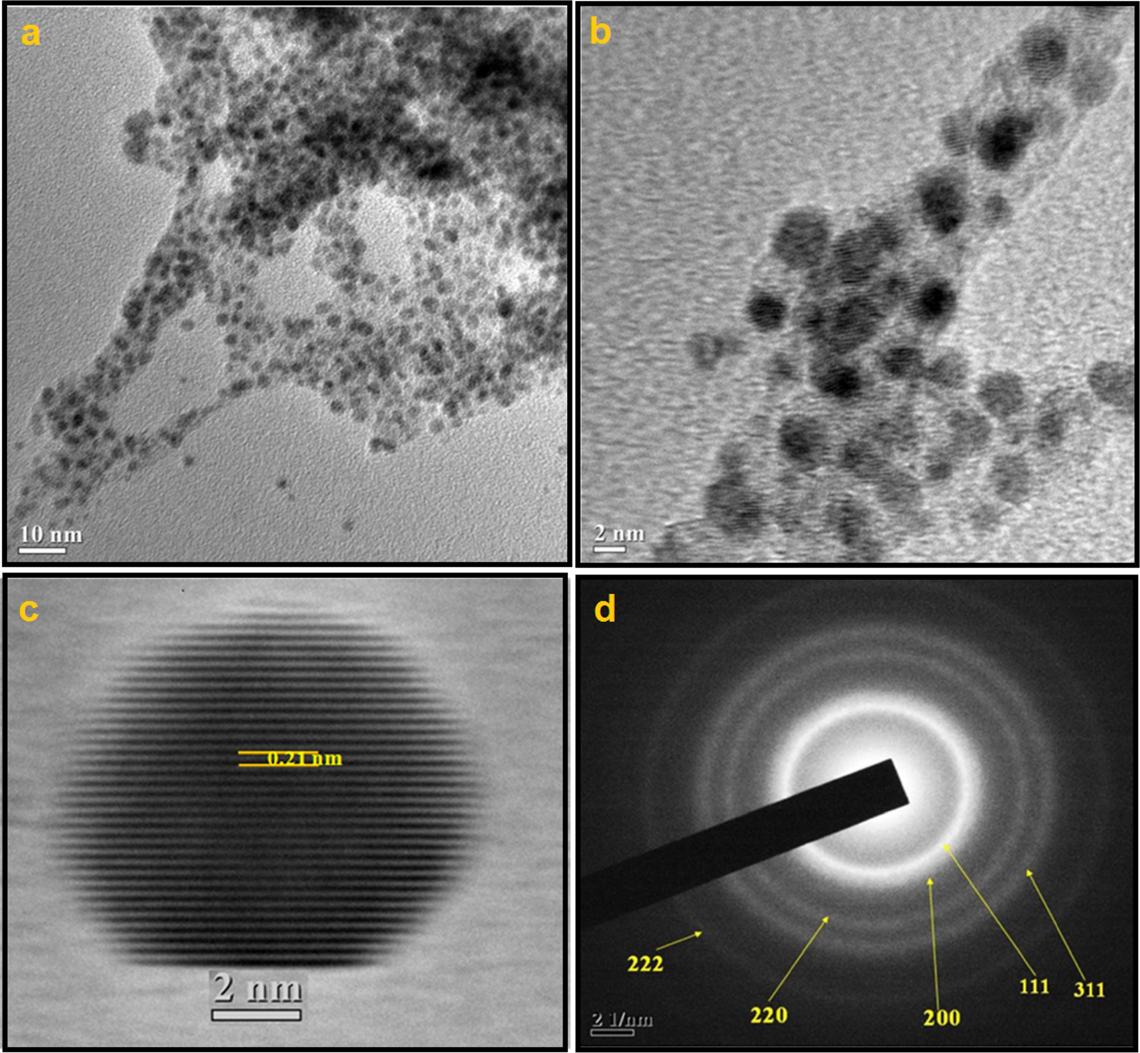
TEM images; (a) & (b) Highly dispersed spherical PdNPs synthesized by using *G*. *pedunculataRoxb*. leaf extract; (c) magnified TEM image of a single metal nanoparticle along with lattice fringes for (111) fcc plane; (d)selected-area electron diffraction (SAED) pattern.

The morphology of the starch-assisted Pd NPs was studied by SEM and TEM analysis. The TEM images reveal that NPs are well dispersed and are of both spherical and non-spherical morphologies with sizes ~2–4 nm (Fig 1A and 1B). Interestingly, when the same synthesis was carried out in absence of starch, the nanoparticles are found to be in the agglomerated form [ESI: S3 Fig].The high magnification TEM image of the Pd NPs along with the lattice fringes with an inter fringe distance of 0.21 nm corresponding to the (111) plane of PdNPs is presented in Fig 1C. The selected area electron diffraction (SAED) pattern (Fig 1D) is consistent with the face centre cubic (fcc) arrangement of the Pd nanocrystals [35].Unfortunately, the Pd particles are not clearly visible in the SEM image, although the EDX analysis shows the presence of Pd along with other elements such as N, O, and S (ESI: S4 Fig). The FTIR spectra of the Pd-based material (ESI: S5A Fig) shows three very prominent peaks at 3338(br), 1616 and 1750 cm^-1^. The first two peaks could be attributed to theυ_OH_stretching andυ_OH_bending of the phenolic hydroxyl groups, while the 3^rd^ peak could be due to the carbonyl group of gallic acid. For comparison, an FTIR spectrum of the powdered dry leaf sample was also recorded (ESI: S5B Fig). In fact, those three peaks of the Pd NPs were also present in the FTIR spectra of dry leaf sample. However, significant shifts of the υ_OH_ stretching (from 3338 cm^-1^ to 3269) and bending (from 1616 to 1652 cm^-1^) were noticed suggesting involvement of a coordinative interaction between the Pd and the galic acid.In addition, some minor changes have also been observed in the far-infrared region. For instance, a band at 384 cm^-1^ in the dry leaf sample shifts towards lower wavenumber upon interaction with Pd.

**Fig 2:**
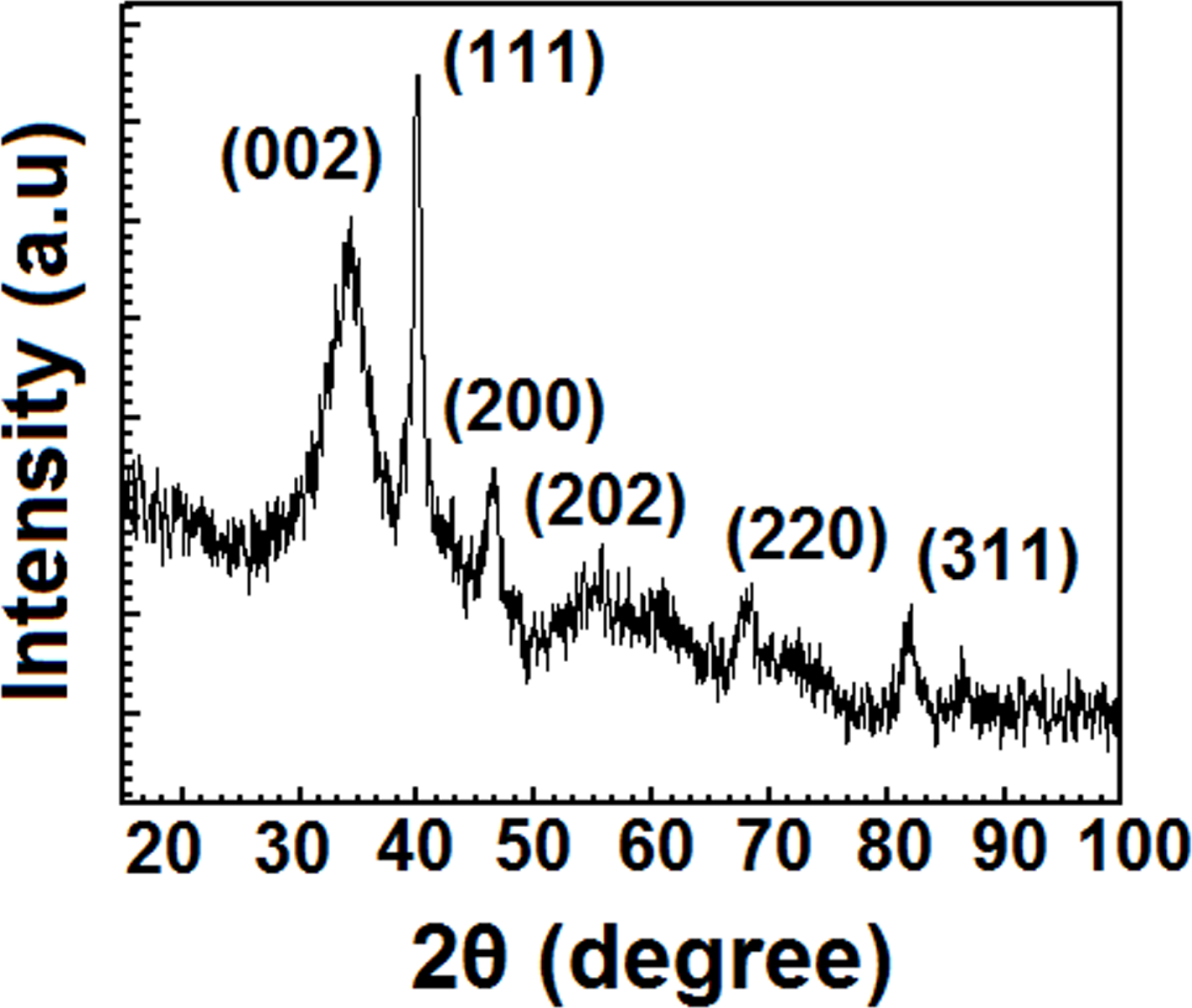
Wide-angle powder XRD pattern of biosynthesized PdNPsin presence of starch (0.3%).

The formation of PdNP was further evidenced from the XRD investigation. The XRD pattern of the Pd NPs is shown in Fig 2. The distinct peaks of the Pd NPs were observed at 2θ of 39.96°, 46.50°, 68.54° and 82.16° diffracted from the (111), (200), (220) and (222) planes with corresponding *d*-spacing values of 2.254, 1.951, 1.37 and 1.172 Å respectively (JCPDS card No. 001–1201). In addition, three other peaks were also observed at 2θ of 34.22°, 55.72°, 86.38° with corresponding *d*-spacing values of 2.62, 1.65, and 1.12 Å indicated the presence of PdO phases along with the Pd NPs (JCPDS card No. 002–1432). It may be noted that often wide-angle XRD issued to investigate size and shape of nanoparticles[36].UsingScherrer equation, the average size of the Pd nanoparticles are found to be about 1.5 nm which is close to that obtained from the TEM spectra. However, when the particle size distribution was measured through DLS, an average hydrodynamic diameter of 322 nm was found; possibly imply aggregation of nanoparticles occurs in the aqueous suspension. Similar types of larger particle sizes determined by DLS study(ESI. S6 Fig) compared to those determined by TEM are also found in literature with other Pd-based materials [37].

**Fig 3:**
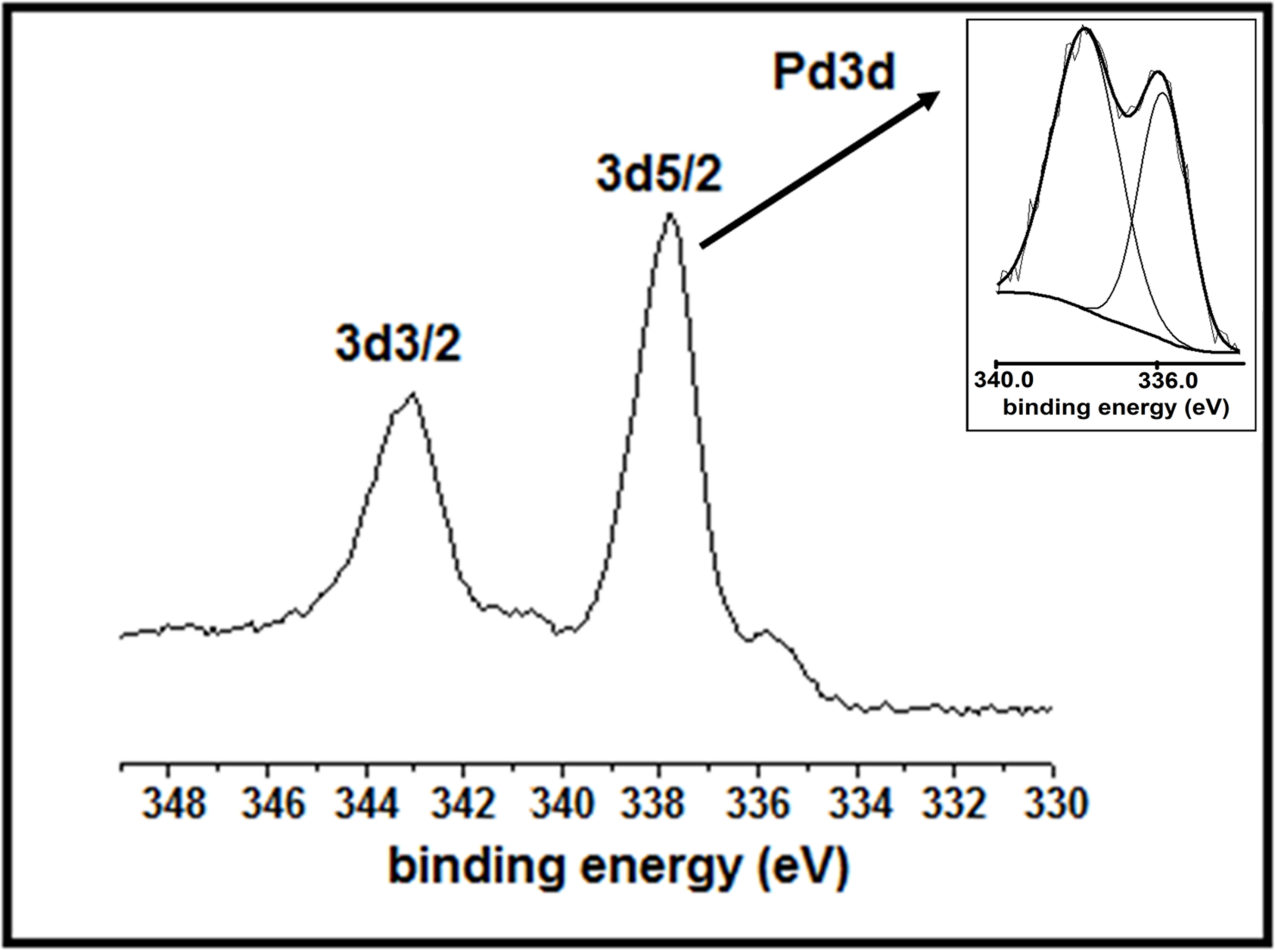
The high resolution spectrum of the starch-assisted PdNPs (Pd 3d region); inset: curve fitted 3d5/2 peak.

The presence of two different oxidation states of Pd was further corroborated by the characteristics 3d3/2 and 3d5/2 peaks in the XPS spectra. The high resolution peak of Pd 3d5/2 (Fig 3) can be deconvoluted into two peaks: a larger one is at 337.8 eV and a smaller one is at 335.7 eV. The first peak is typical for a Pd(II) species, while the second one is for Pd(0). The XPS survey spectrum of the material shows in addition to palladium, the material also contain carbonand oxygen From their atomic percentages, the surface amount of Pd was calculated as 1.20 mM/g. The O1s peak (ESI. S7 Fig) appears at 533.1 eV which is very near to that reported for PdO [38,39].The thermal decomposition study of the bio-synthesized PdNPs indicated a three-step degradation process (ESI: S8 Fig). The initial minor decay of about 1.5% at around 100°C was attributed to phsyi-adsorbed water associated with the nanomaterials, while the second and third decay at 235–345°C and 345–682°C with corresponding weight lossesof 69% and 23% could be attributed to the phenolic–OH and the bio-organic molecules respectively. Residual weight of14% could be ascribed to metallic Pd or PdO.

### Suzuki-Miyaura activity

**Fig 4:**
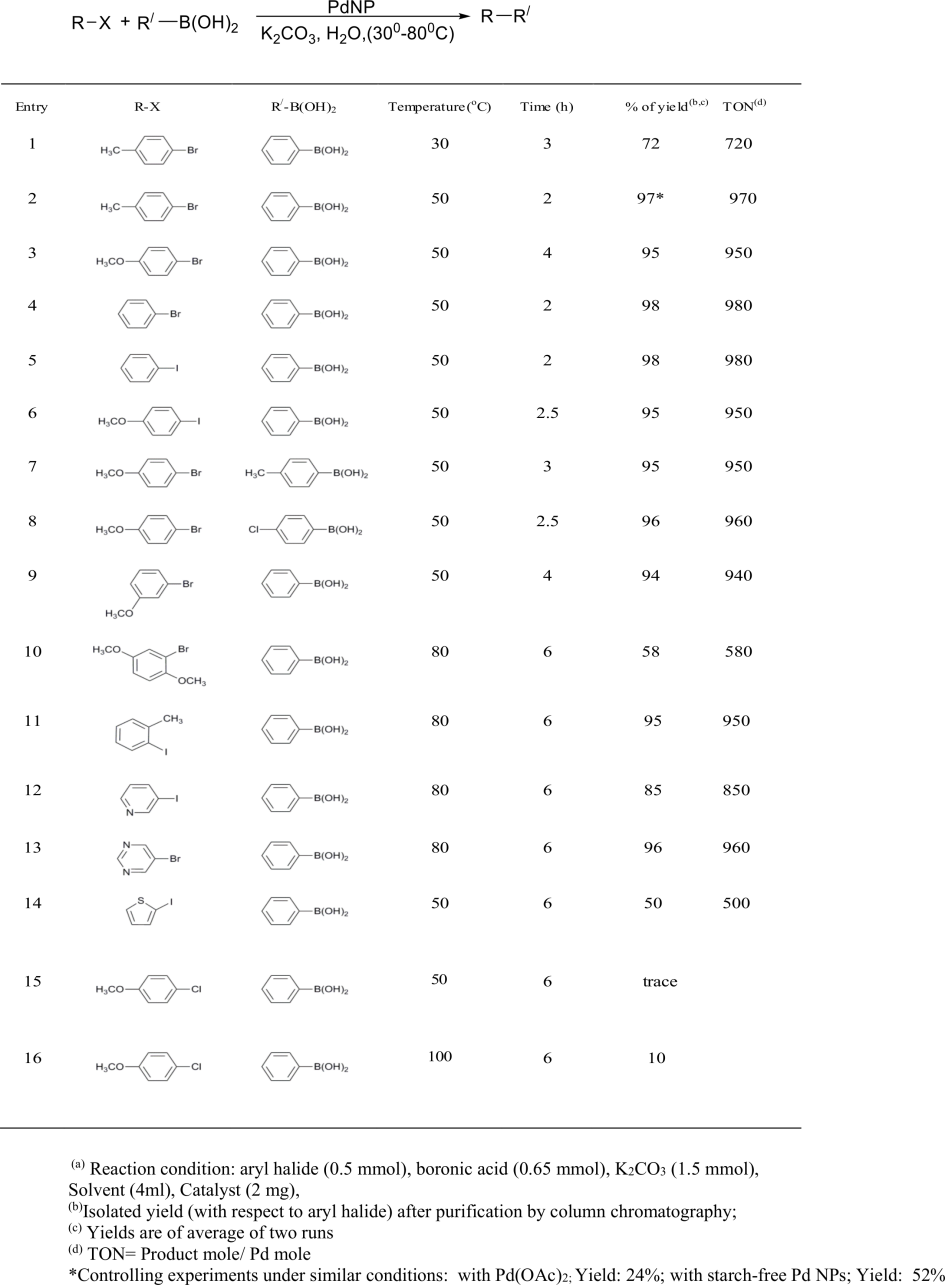
Suzuki-Miyaura reactions^a^ of various aryl/heteroaryl halides with arylboronic acids using PdNPs as catalyst.

The palladium catalyzed Suzuki-miyaura cross-coupling reactions between aryl halides and aryboronic acids is one of the most extensively studied reactions in organic synthesis [3,40]. A wide range of Pd-catalysts including Pd NPs are known that could perform this reaction under mild conditions. It is worth to note that PdNPs of less than 5 nm often show very good catalytic activities in the Suzuki-Miyaura cross-coupling reaction[41–43]. To check the catalytic activity of the synthesized PdNPs, we have preformed the Suzuki-Miyaura reaction using *p*-bromotolouene and phenylboronic acid as model substrates. Initially, the model reaction was performed at room temperature in water with 2 mg of catalyst using K_2_CO_3_ as base and 72% (Fig 4, entry 1) product formation was achieved after a span of 3h. However, increasing the temperature to 50°C, almost quantitative formation of biphenyl was obtained within 2h (entry 2). Based on our initial success; we have tested the scope of our catalytic system for other representative aryl bromides or iodides including heteroaryl halides that are usually difficult to activate in the Suzuki reaction. Our study revealed that aryl bromides and iodides bearing electron-neutral (entry 4 & 5), electron-withdrawing (entry 3 & 6) and electron-donating groups (entry 2) underwent smooth coupling with phenylboronic acid and almost quantitative conversion was obtained in all the cases. Interestingly, this high yields were also maintained when phenylboronic acid was replaced with *p*-tolyl- or *p*-chloroboronic acid (Entry 7 & 8). Moreover, at an elevated temperature (80°C) and with an extended reaction time our system can also tolerates difficult substrates like sterically demanding 2-iodotoluene (entry 10) and 1-bromo-2,5-dimethoxybenzene (entry 11), or heteroaryl halides like 3-iodopyridine (entry 12) or 5-bromopyrimidine (entry 13) and provided the cross-coupling products in good-to-excellent yields.It needs to mention that aturnover number (TON) close to 1000 was obtainedwith our catalyst for cross-coupling with a few aryl bromides in water,and this value is either comparable[44] or superior [45]with some of the reported PdNPs-based catalysts that are chemically synthesized. Unfortunately, despite good results with aryl bromides or aryl iodides, our catalyst is not suitable for activating aryl chlorides as substrate (Entry 15 & 16).Noteworthy to mention that usually aryl chlorides are difficult substrates to be activated in Suzuki reaction because of stronger C-Cl bond compared to C-Br or C-I bond [9].

**Fig 5:**
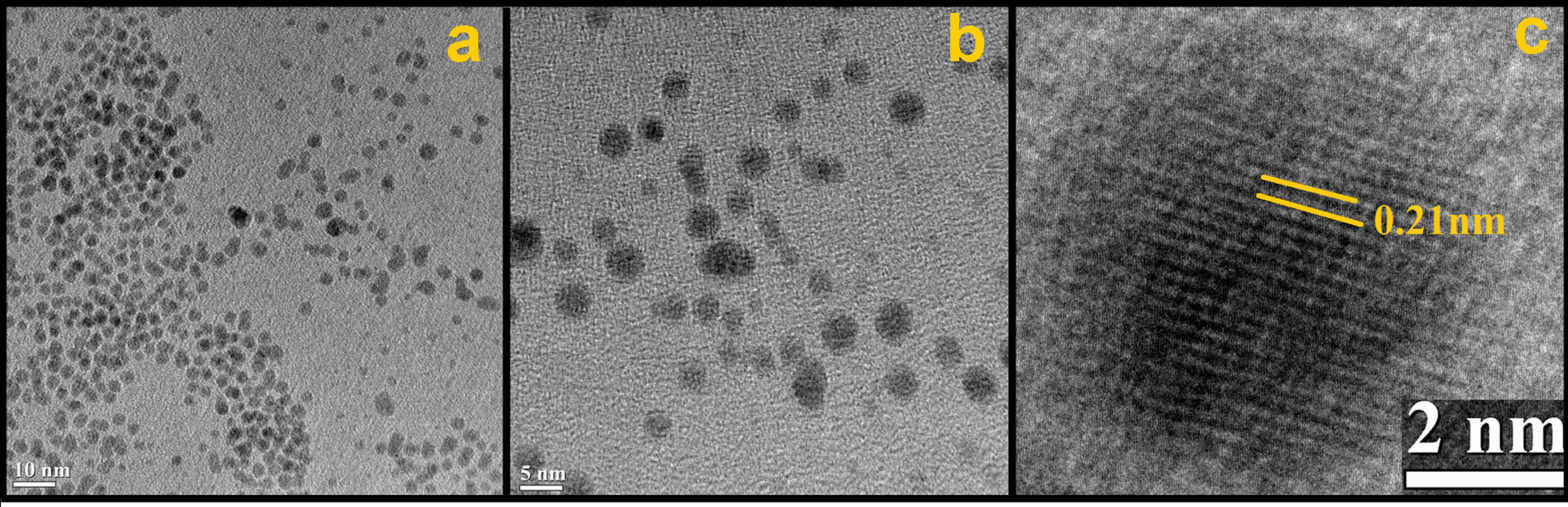
TEM images of the recycled catalyst showing lattice fringes corresponds to (111) plane of Pd NP.

It may be important to note that one of the key advantages of NPs based catalytic system is their potential recyclability, and to check this property, we have performed the reusability experiment with our model system taking *p*-bromotoluene and phenylboronic acid as model substrate Table 1. For handling convenience, we have performed the reaction with 10 mg catalyst using proportionate amount of substrates, base and water. Our study revealed (Fig 4) the nanocatalyst could be reused at least for four cycles; however a gradual decrease in yield was observed which might be due to the handling loss of the catalyst during centrifugation. The TEM pictures (Fig 5) of the reused catalyst showed almost similar morphology with no sign of aggregations suggesting that the catalyst could be reused for further runs.

**Table 1:** Recycling of the catalyst for the reaction between 4-bromotoluene and phenylboronicacid^a^.

Cycle	Yield^b^
1	95
2	92
3	91
4	89

^a^Reaction condition: 4-bromotoluene (2.5mmol), phenylboronic acid (3.25 mmol), K_2_CO_3_ (7.5 mmol), H_2_O (20 mL), Pd catalyst (10 mg); 50°C

^b^Isolated yield.

### Alcohol oxidation reaction

**Fig 6:**
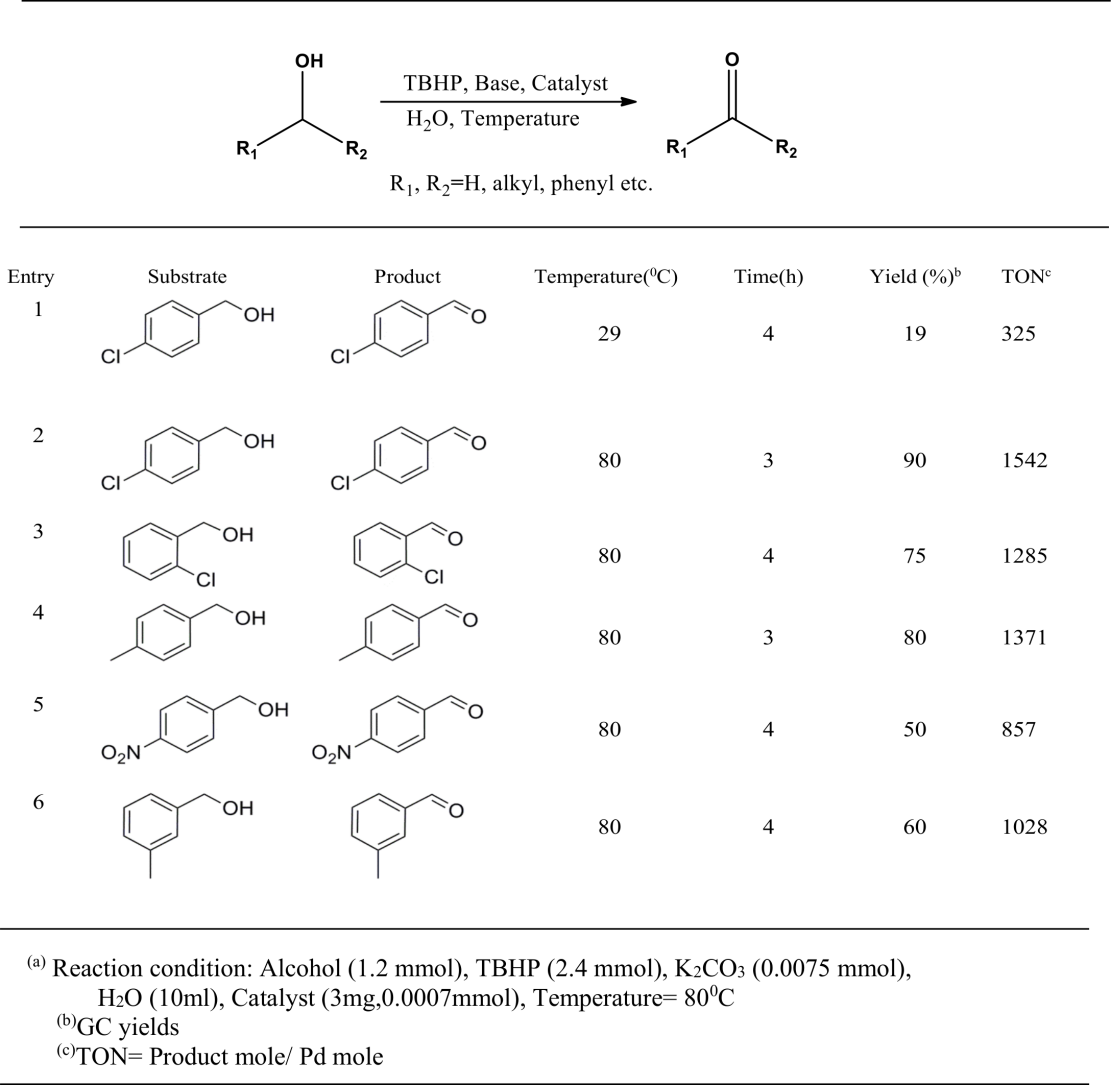
Oxidation of selective alcohols in aqueous media catalyzed by PdNPs as catalyst.

Oxidation of alcohols to carbonyl compounds is one of the most important reactions in organic synthesis. Palladium-based catalysts, including Pd NPs,are particularly known to promote this reaction under mild conditions using environmentally benign oxidants such as O_2_, H_2_O_2_, *tert*-butyl hydrogen peroxide (TBHP), *etc* [11,46]. However, in the majority of the cases, the reactions are usually conducted in organic solventsthat are not only toxic but also expensive. With the bio-synthesizedPd NPs at hand, we have expanded the scope of the catalyst for alcohol oxidation reactions using water as solvent. Initial study was performed at room temperature using *p*-chlorobenzylalcohol as model substrate withTBHP as oxidant in presence of K_2_CO_3_ as base using 3 mg of the Pd NPs. After a reaction time of 4h, only 19% *p*-chlorobenzaldehyde was formed. However, on increasing the temperature to 80°C, nearly quantitative yield of product was obtained in 4h. Encouraged by this result, we have tested some more representative alcohols for the oxidation reaction. Alcohols bearing electron-withdrawing groups like *p*-nitrobenzylalcohol (Fig 6, Entry 5) or electron-donating groups like *p*-methybenzylalcohol (Fig 6: Entry 4) could be converted to corresponding aldehydes with moderate to high yields. Our results showed that the position of substituent also have certain impacts on the performance of the catalyst. For instance, oxidation of *o*-chlorobenzylalcohol gave 75% yield, while under similar condition *p*-chlorobenzylalcohol gave 94% yield.

### Reduction of Chromium(VI) to Chromium(III)

**Fig 7:**
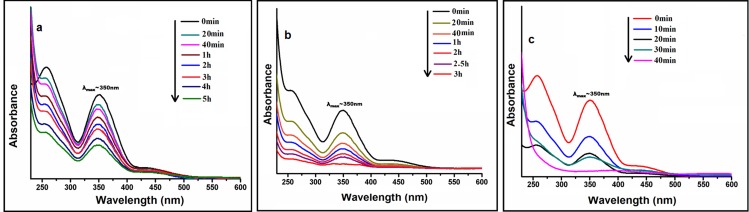
Cr(VI) reduction: (**a)** in absence of catalyst; (b) in presence of Pd(OAc)_2_ salt; (c) in presence of starch-assisted Pd NPs as catalyst.

Chromium(VI) compound is considered to be one of the major pollutantscommonly found in ground water at the industrial sites [47]. At present, various remediation strategies are available that can detoxify chromium, and one such method is the catalytic reduction of Cr(VI) to Cr(III). Among various catalysts, NP-based systemsincluding Pd NPs are found to be particularly promising [48]. To investigate the effectiveness of our catalyst for this reaction, an aqueous solution of K_2_Cr_2_O_7_ was treated with bio-synthesized Pd NPs in presence of formic acid as reducing agent and the degradation of Cr(VI) to Cr(III) was monitored with UV-Vis spectrophotometer. The aqueous solution of K_2_Cr_2_O_7_ exhibits a strong peak at 350 nm which gradually decreases with time (Fig 7). Upon stirring the reaction for 40 min. this peak finally vanishes indicating complete reduction of Cr(VI) to Cr(III) and the light yellow colour of K_2_Cr_2_O_7_ solution changed to colourless. It is worth noting that under the same set of experimental conditions, K_2_Cr_2_O_7_ and Pd(OAc)_2_ showed only 17 and 57% degradations respectively.

### Evaluation of anti-microbial activity against MDR isolate

**Fig 8:**
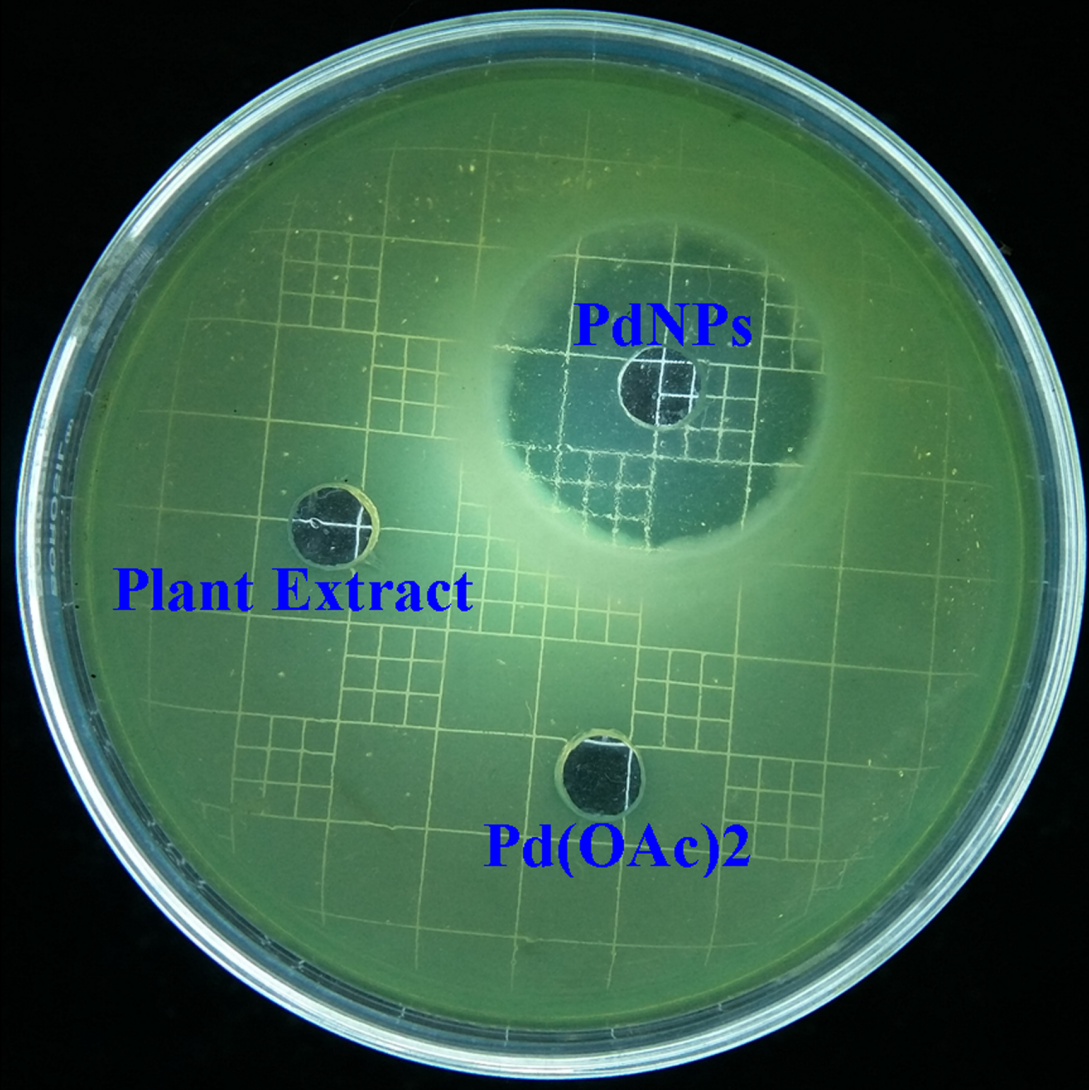
Figure showing clear zone of inhibition around the well containing Pd NPs confirm its antimicrobial activity against MDR *Cronobactersakazaki* AMD04 strain; whereas the aqueous plant extract (PE) shows no effect and Pd(OAc)_2_ shows minor effect against the isolate.

**Fig 9:**
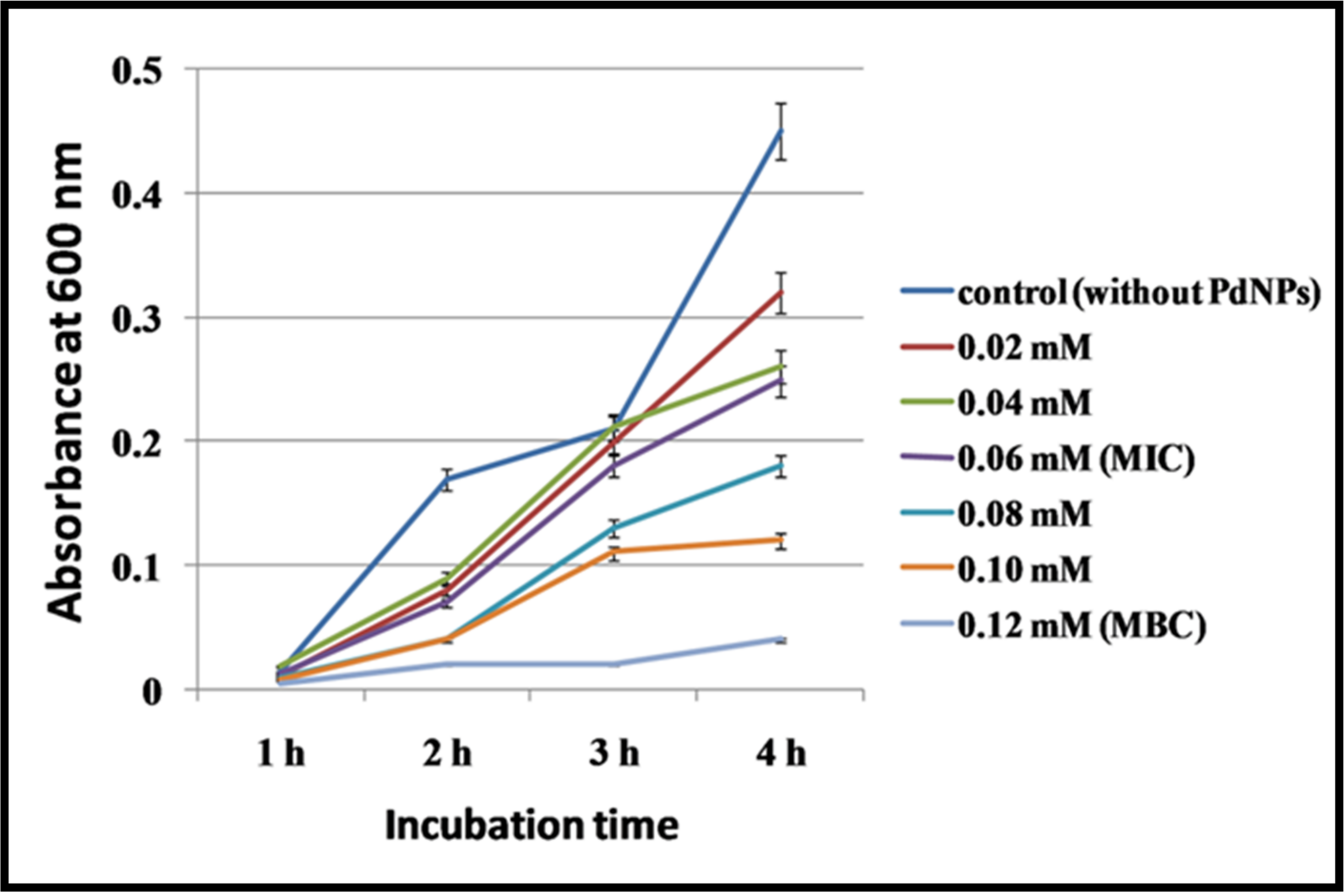
Graph showing MIC and MBC of PdNPs against *Cronobactersakazakii* strain AMD04 at different time intervals.

The most potential MDR bacterial isolate (*i*.*e*. showing resistance against maximum number of antibiotics *viz*., ampicillin, imipenem, tetracycline, and rifampicin) was identified as *Cronobactersakazakii*strain AMD04 by 16S rDNA sequencing technique. The NCBI Gene Bank accession no. KJ812198.1 was received for the novel isolate (ESI.S9 Fig). Antimicrobial activity of PdNPs was carried out by agar well diffusion method. Clear zone of inhibition with a diameter of 31.67±1.53 mm around the well containing PdNPs in bacterial culture plate clearly confirm its bactericidal effect against the isolates (Fig 8). Whereas, aqueous plant extract and Pd(OAc)_2_ solution show no effect against the isolate. The PdNPs exerted an MIC and MBC of0.06mMand0.12mMrespectively against *Cronobactersakazakii* AMD04 (Fig 9) which was measured spectrophotometricallyat an absorbance of 600 nm. It may be noted that although bacterial growth can be measured at any of the visible light range, measuring the 600 nm peak is a standard method of monitoring growth, as this wavelength corresponds to orange colour and most bacterial cultures tend to grow orange as their culture grows more dense[49].These MIC and MBC values aremuch lower than FDAoral tolerable daily intake for dosage forms and components (*i*.*e*. 100μg/day) [50]. This finding makes PdNP a potential candidate for using as a drug component to fight against MDR bacteria.

## Biofilm inhibition activity by PdNPs

**Fig 10:**
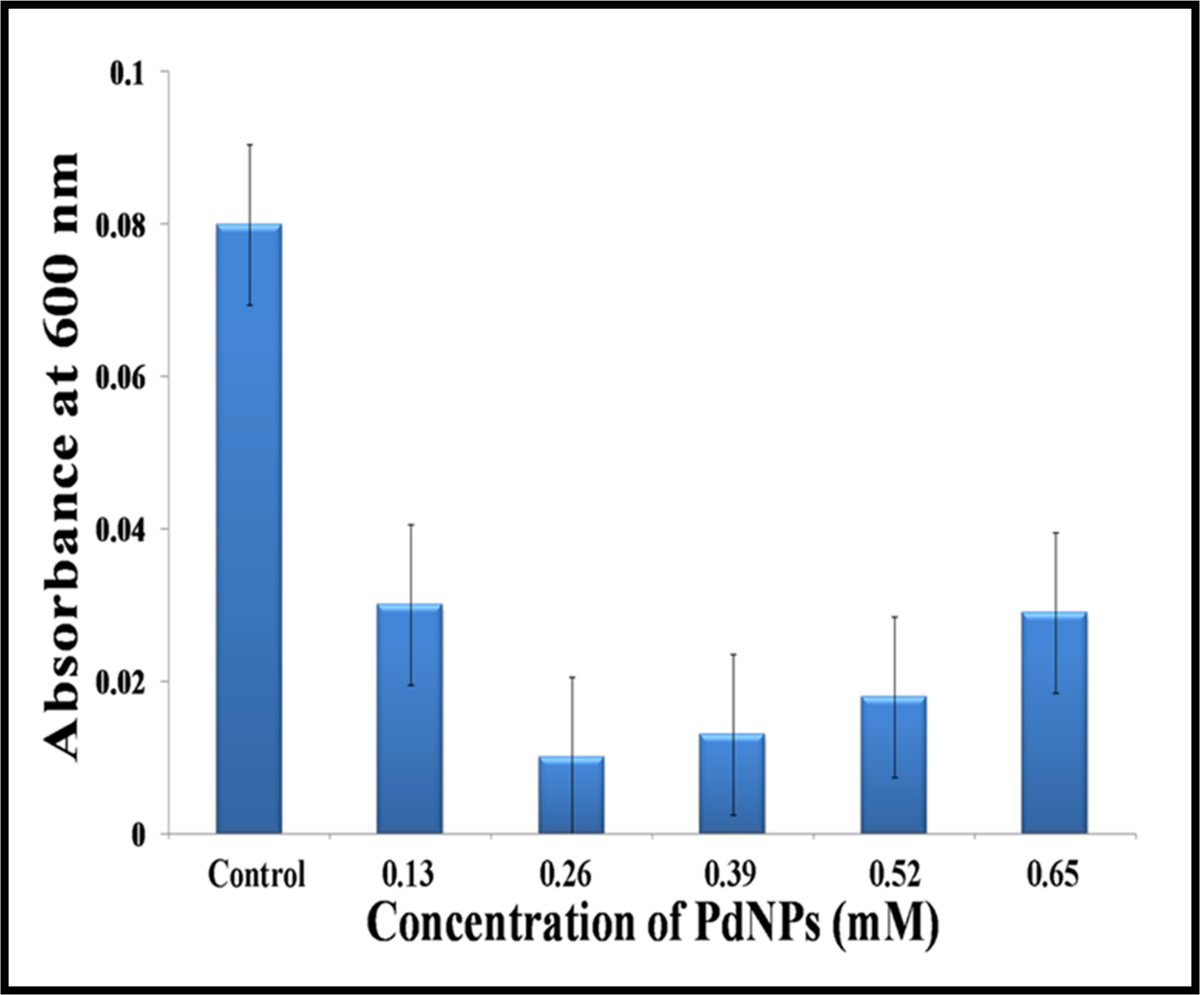
Graph showing anti-biofilm effect of PdNPs against MDR clinical isolate *Cronobactersakazakii* strain AMD04.

Antibacterial activity with chemically synthesized Pd NPs has been previously reported[35], although reports on biofilm inhibition activity by Pd NPs have not been seen so far.We are intrigued to see whether our biosynthesized PdNPs are able to eradicate biofilms which are known to display high resistance to toxic doses of antimicrobial agents that usually eradicate planktonic cells. It may be noted that inhibitory effects of metal nanoparticles (e.g. AuNPs, [51] ZnO-NP[52], etc) on pathogenic biofilm formation is an active area of research that has got many recent attentions. When tested the PdNPs against novel MDR clinical isolate *Cronobactersakazakii*strain AMD04, it displayed significant decrease of biofilm biomass. The experiment was done in triplicate and results are expressed in terms of mean ± S.D. Maximum inhibition of biofilm was recorded at low concentration (0.26±0.02 mM) of PdNPs (Fig 10). Comparable decrease in biomass formation was also observed at 0.39mM and 0.52mM concentrations. At concentrations below 0.26mM and above 0.65mMPdNP, *Cronobactersakazakii* exhibited slight increase in absorbance. This increase in absorbance is due to the interference caused by increased concentration of PdNPs. Most unremitting and relentless bacterial infections are associated with biofilm growth, a strategy that has accelerated the emergence and hasty spread of multidrug resistant bacteria. It is well known that biofilm associated bacteria are much more difficult to be eradicated by traditional bactericidal antimicrobials than planktonic cells. Moreover the PdNPs exhibited their potential as biofilm inhibitor against novel MDR clinical isolate *Cronobactersakazakii*AMD04.On the basis of literature report [51], a proposed mechanistic pathway of biofilm inhibition by Pd NPs has been shown in (ESI.S10 Fig). Planktonic bacterial cells adsorb on the substrate and attaches with each other by cell signaling and subsequent release of exopolysaccharide (EPS) forming a protective matrix. Similar studies carried out using AuNPs suggested that the increased stress exerted by nanoparticles on microbial cells resulted in increased planktonic growth and decreased adhesion of microbes on substrate.

## Conclusion

In summary, we presented a green protocol of synthesizing PdNPs by using aqueous leaf extract of *Garciniapedunculata*Roxb as bio-reductant. The PdNPs showed excellent activity as catalyst for three important reactions namely the Suzuki-Miyaura cross-coupling reaction, alcohol oxidation and reduction of Cr(VI) to nontoxic Cr(III). The Pd NPs also showed excellent antimicrobial and anti-biofilm activities against a novel MDR clinical isolate *Cronobactersakazakii* AMD04. The MIC and MBC values of Pd NPs are much lower than that of USP administration limit for oral tolerable daily intake, which make these PdNPs potential drug candidate to fight against MDR bacteria. The multifunctional applications along with ‘in water’ reactions for both synthesis and catalysis are the main features of our catalyst.

## Supporting information

S1 Fig *Garcinia pedunculata* roxbleaves used for nanoparticle synthesis. (TIF)

S2 Fig HRMS spectrum of bio-extract of *Garcinia pedunculata* Roxb. (TIF)

S3 Fig TEM images of PdNPs in absence of starch. (TIF)

S4 Fig SEM-EDX images of the PdNPs. (TIF)

S5 Fig FTIR spectra of PdNPs andpowdered dry leaf of *Garcinia pedunculata* Roxb. (TIF)

S6 Fig DLS spectra of PdNPs. (TIF)

S7 Fig XPS survey spectrum of the PdNPs. (TIF)

S8 Fig TG thermogram of biosynthesized PdNPs. (TIF)

S9 Fig Phylogenetic relationship of the MDR isolate *Cronobacter sakazaki* strain AMD04 (highlighted in yellow colour) with ten most closely related strains based on BLAST results taking *Erwinia amylovora* ATCC 15580 as out group. (TIF)

S10 Fig ^1^H NMR spectra of the products for the Suzuki-Miyaura reaction. (PDF)

S11 Fig Mass spectra of selected products for the Suzuki-Miyaura reaction. (PDF)

S12 Fig Mass spectra of products for the alcohol-oxidation reaction. (PDF)
