# Supplementary figures and images for: Biogenic synthesis of palladium nanoparticles and their applications as catalyst and antimicrobial agent
Source: PLoS One. 2017 Sep 28;12(9):e0184936. doi: 10.1371/journal.pone.0184936 (PMC5619764; doi:10.1371/journal.pone.0184936)

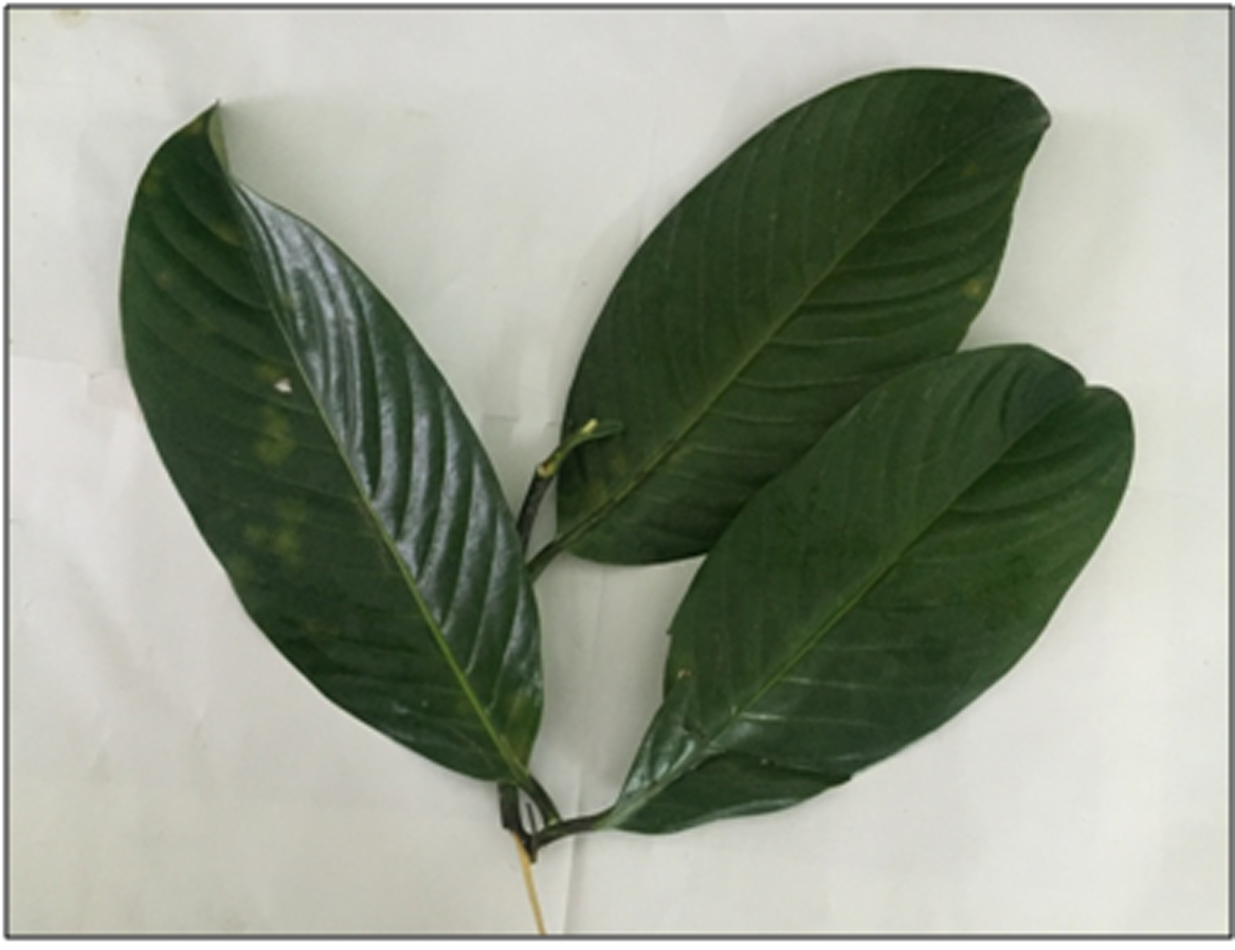

Supplement: S1 Fig — (TIF) [file pone.0184936.s001.tif]

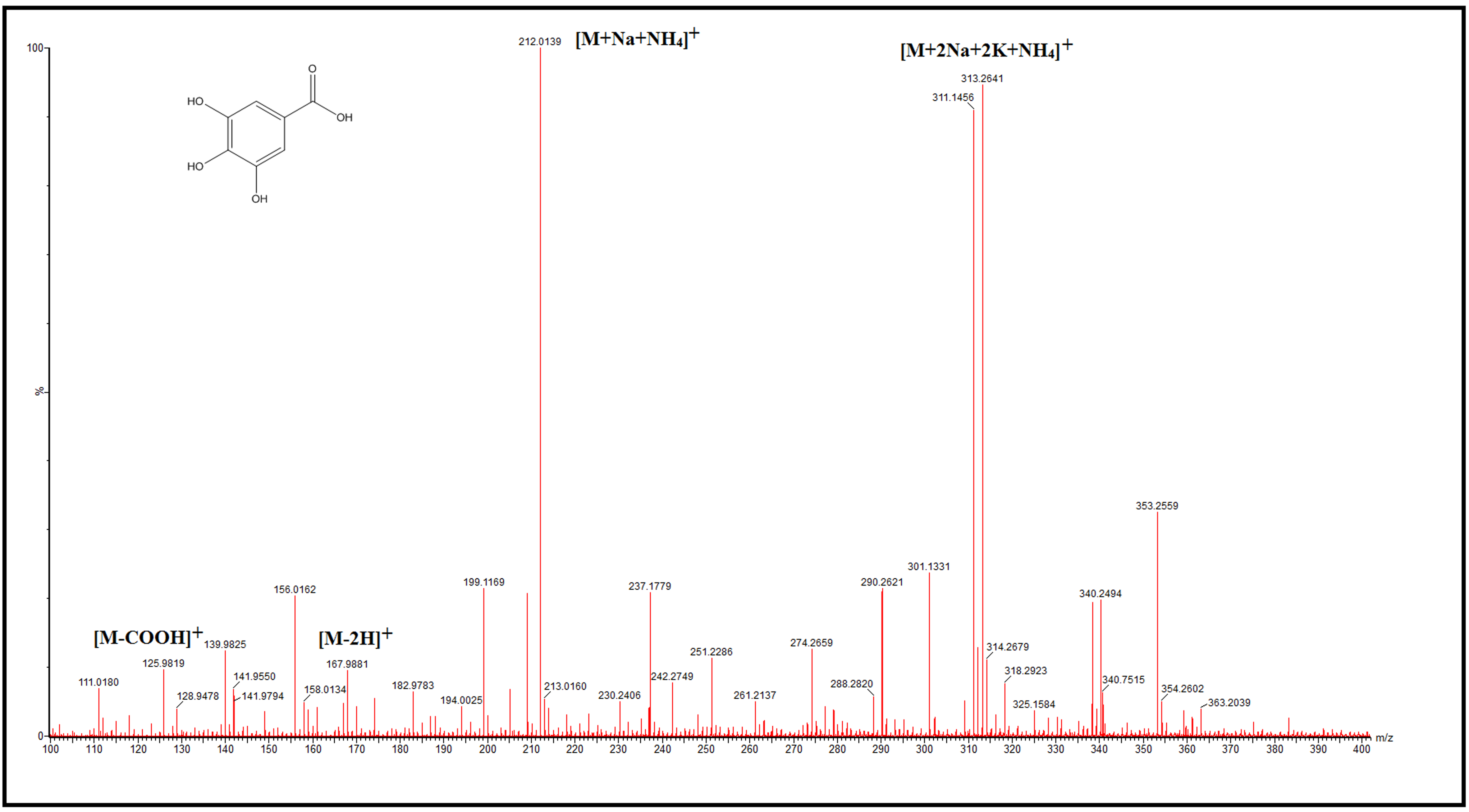

Supplement: S2 Fig — (TIF) [file pone.0184936.s002.tif]

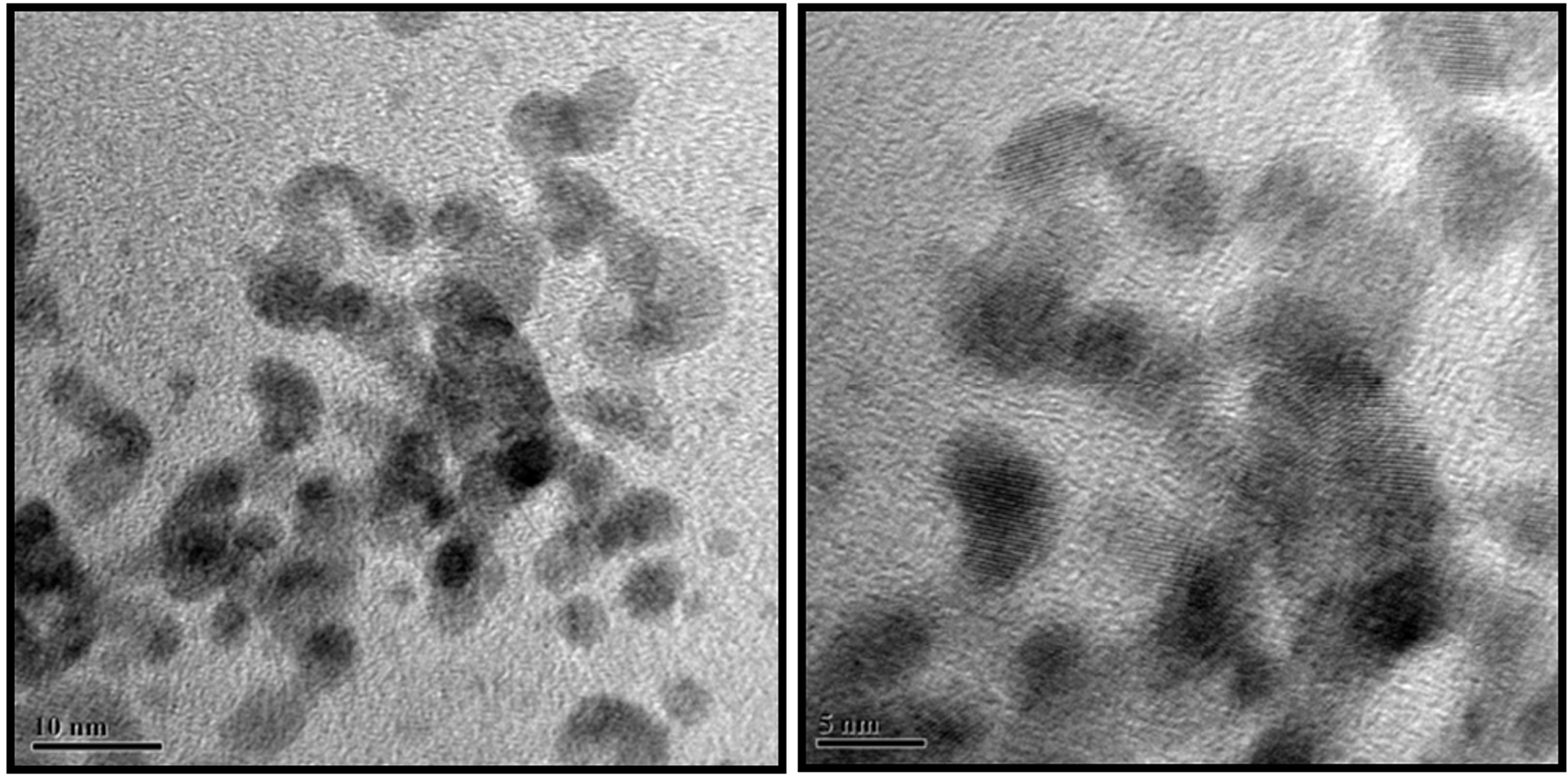

Supplement: S3 Fig — (TIF) [file pone.0184936.s003.tif]

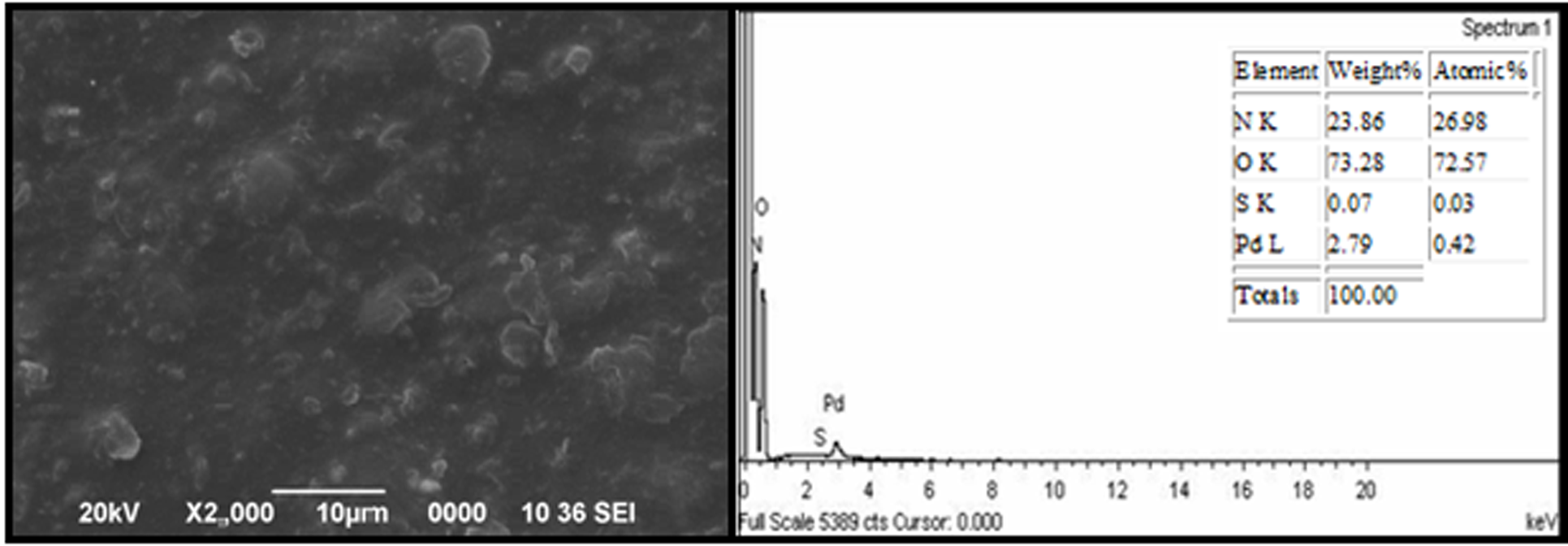

Supplement: S4 Fig — (TIF) [file pone.0184936.s004.tif]

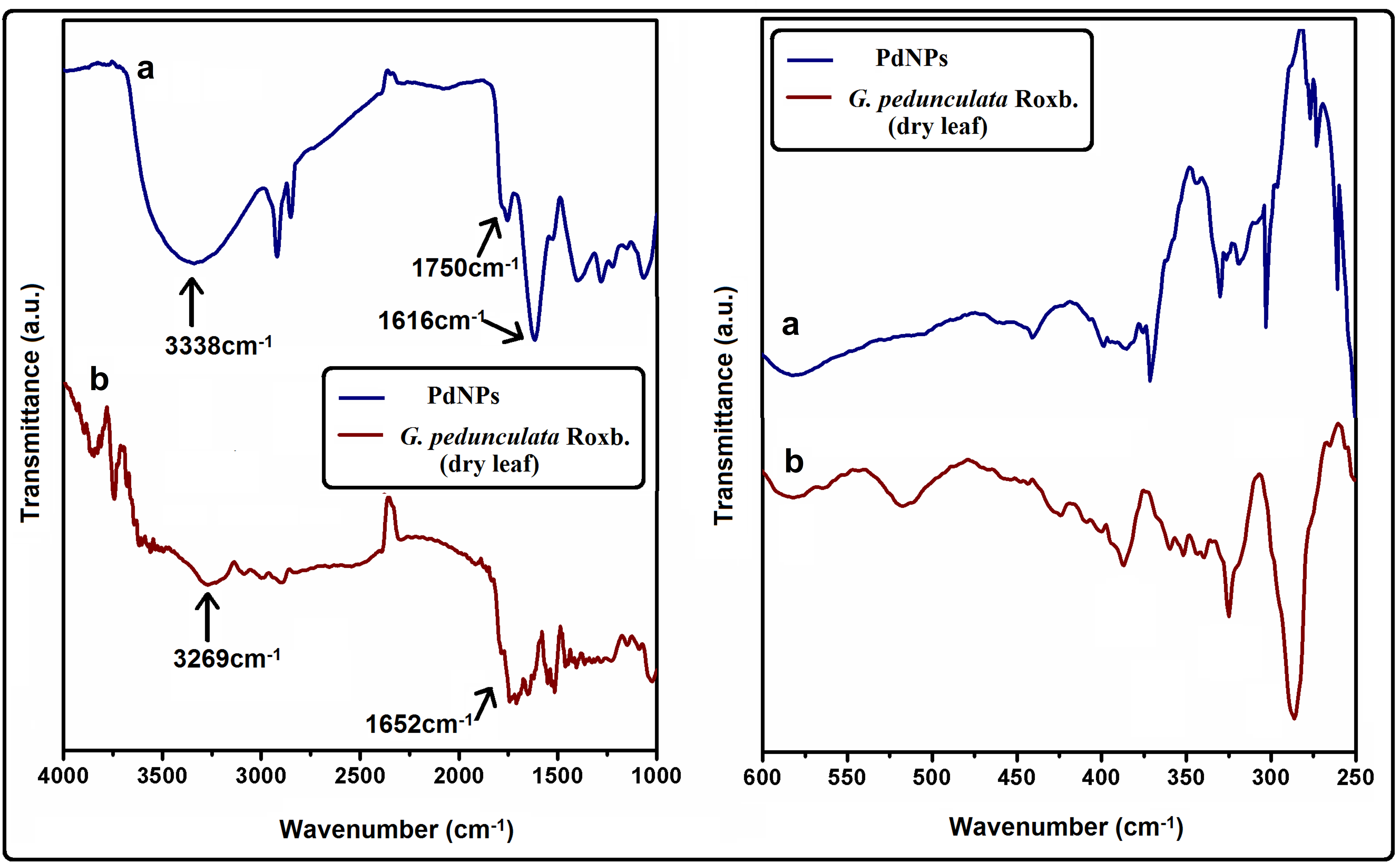

Supplement: S5 Fig — (TIF) [file pone.0184936.s005.tif]

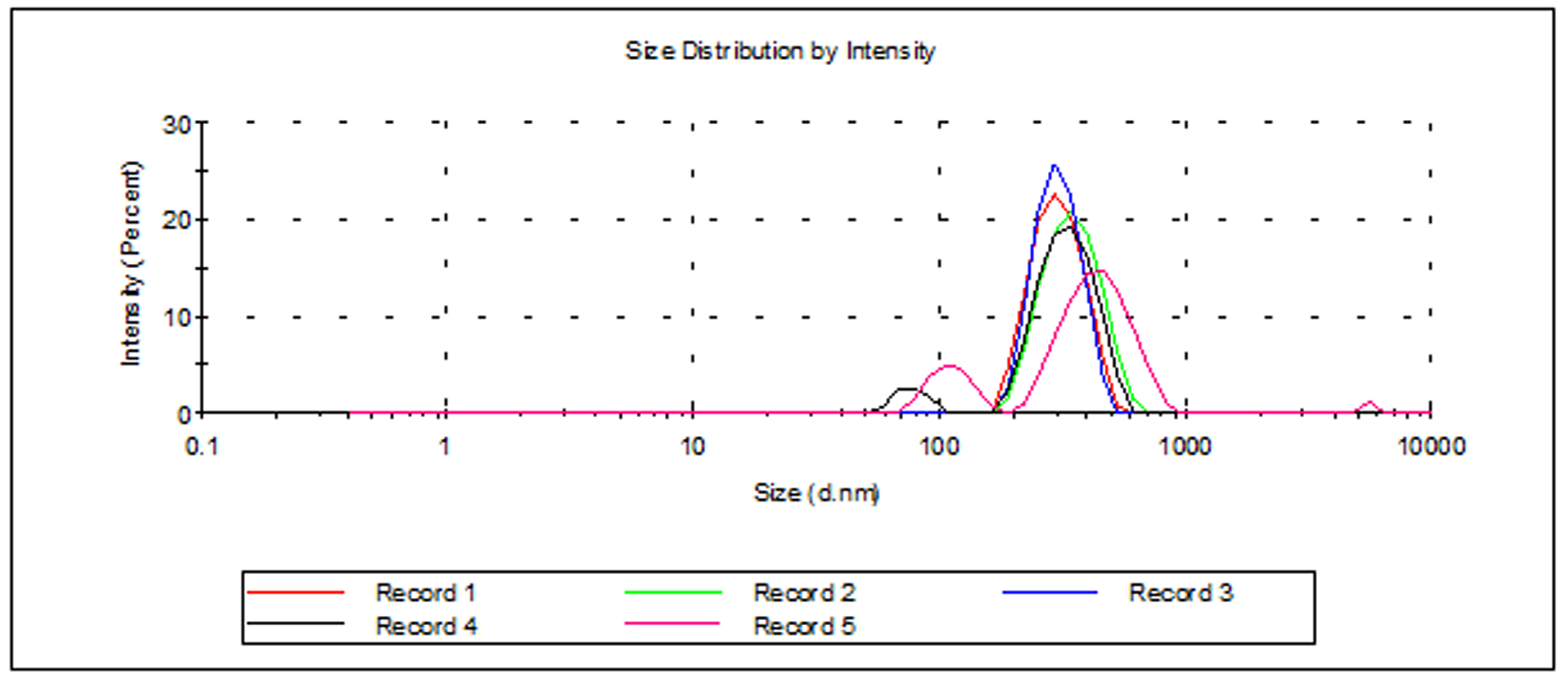

Supplement: S6 Fig — (TIF) [file pone.0184936.s006.tif]

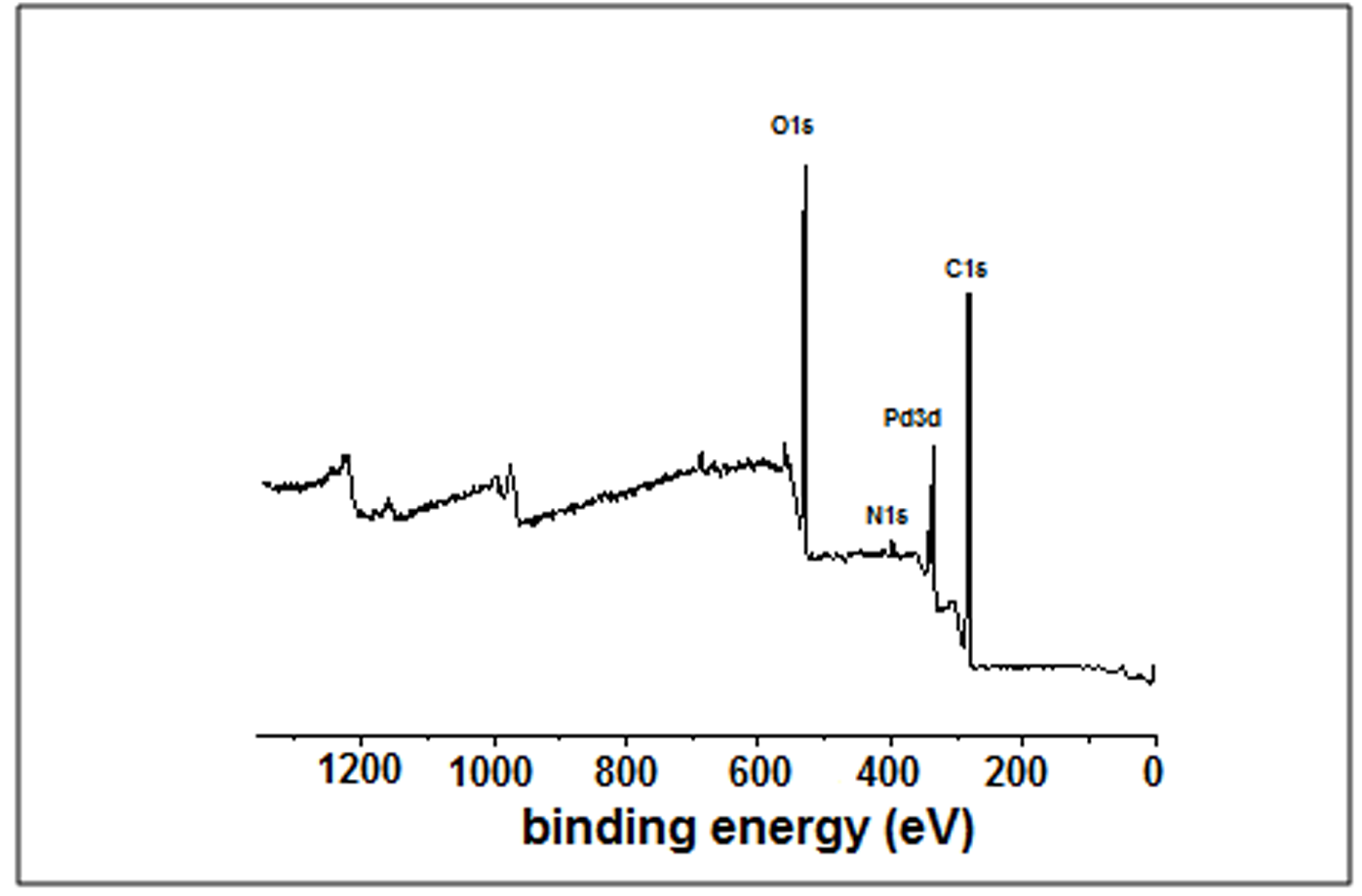

Supplement: S7 Fig — (TIF) [file pone.0184936.s007.tif]

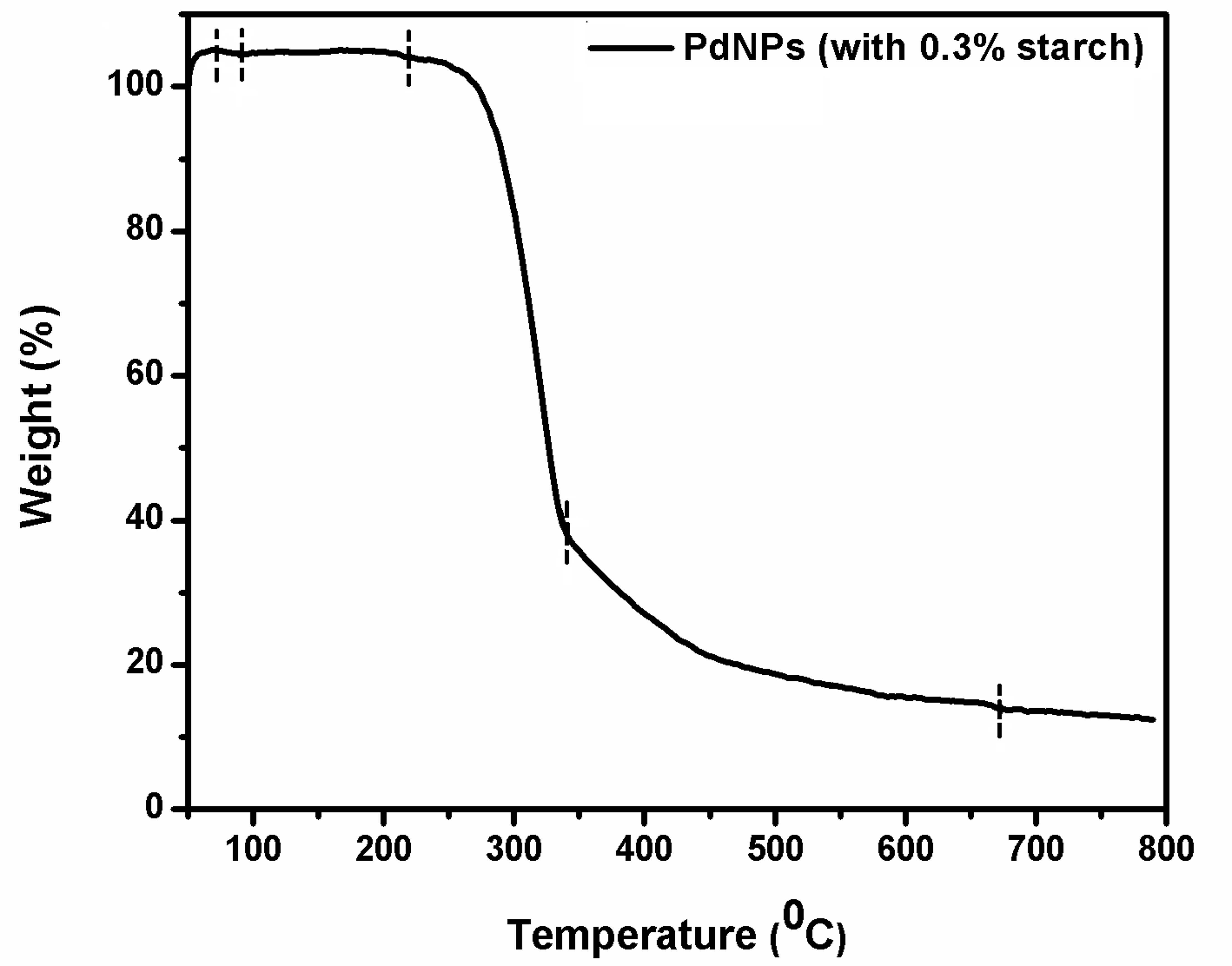

Supplement: S8 Fig — (TIF) [file pone.0184936.s008.tif]

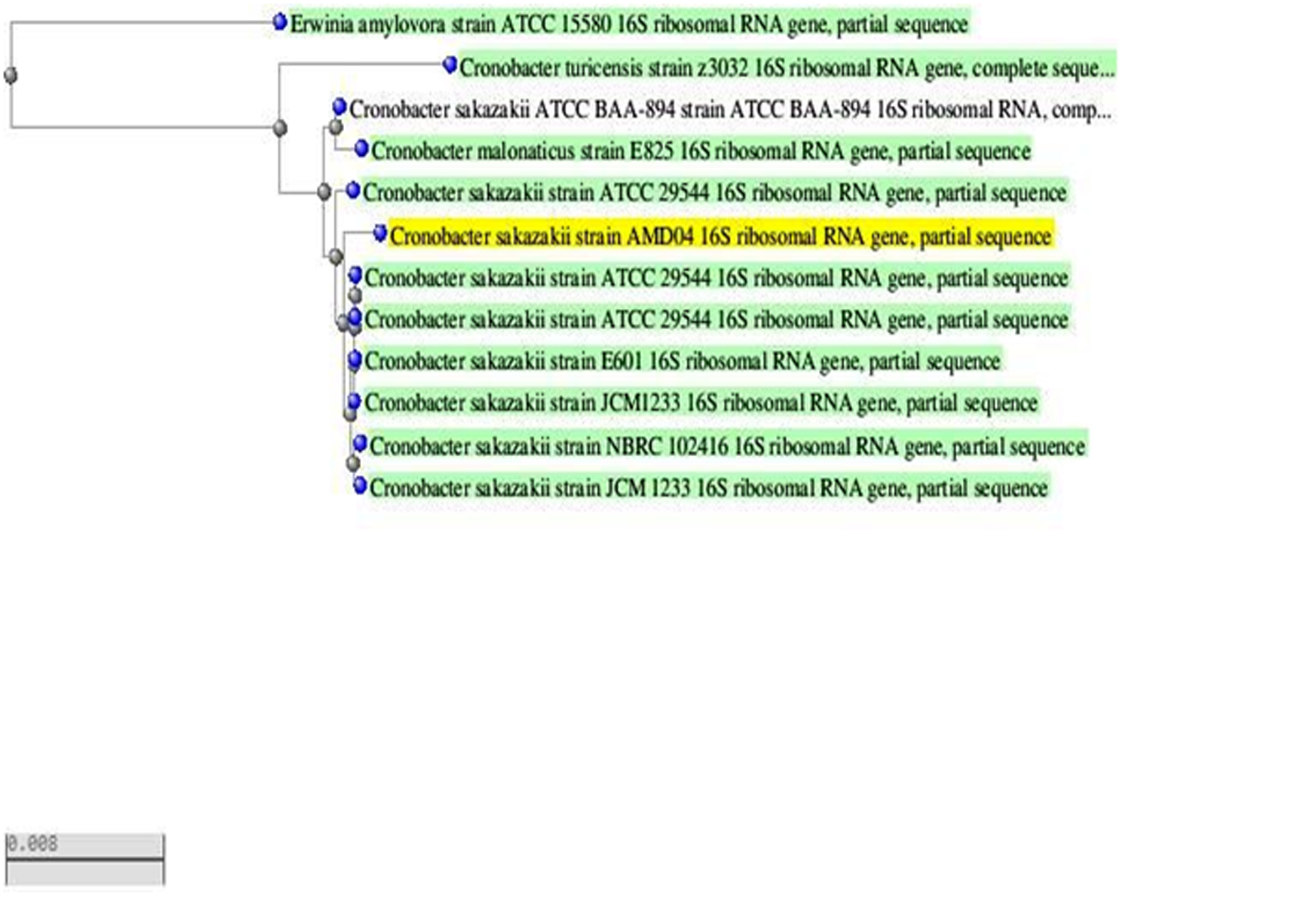

Supplement: S9 Fig — (TIF) [file pone.0184936.s009.tif]

**S10 Fig.**  $^1\text{H}$  NMR spectra of the products for the Suzuki-Miyaura reaction

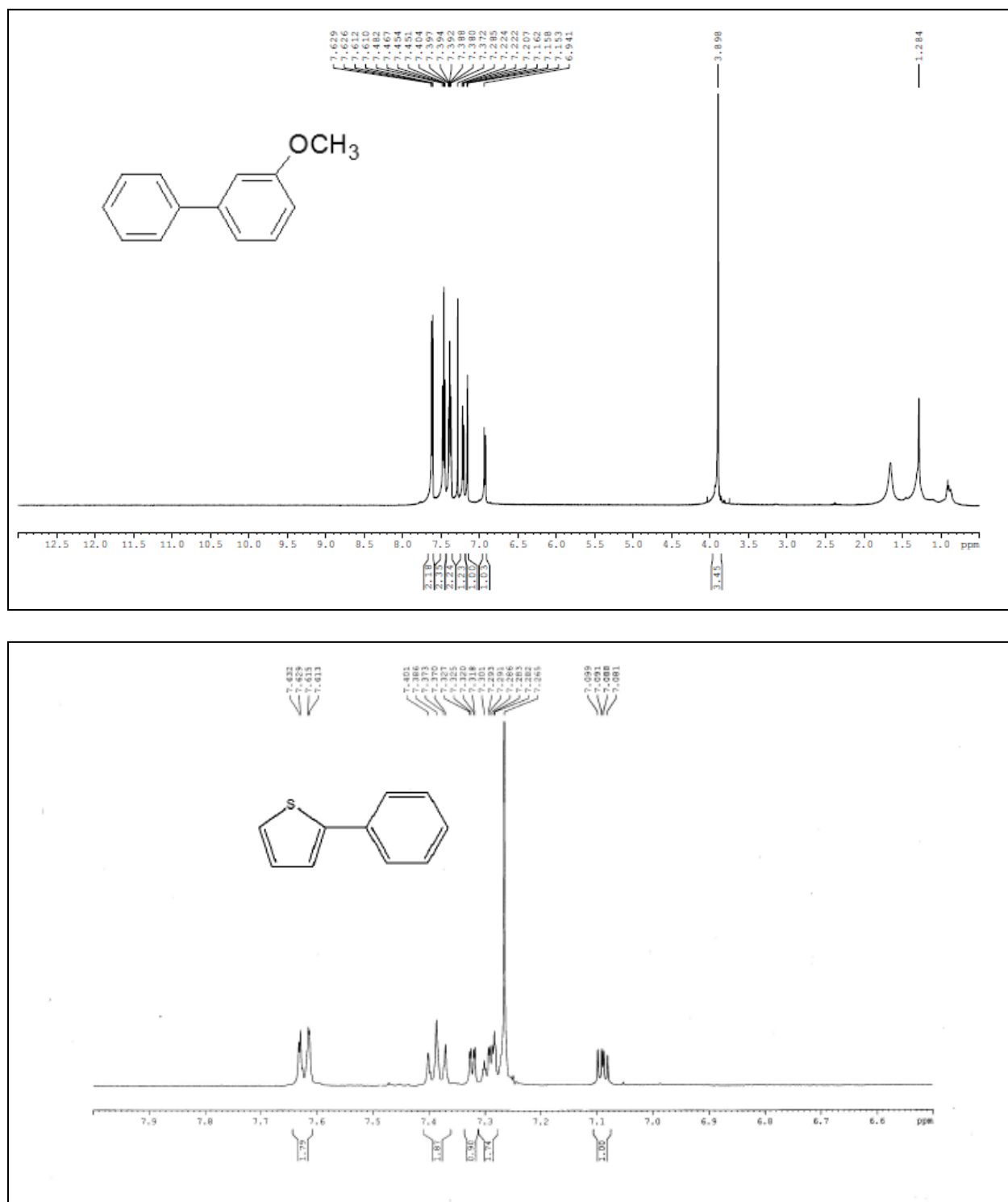

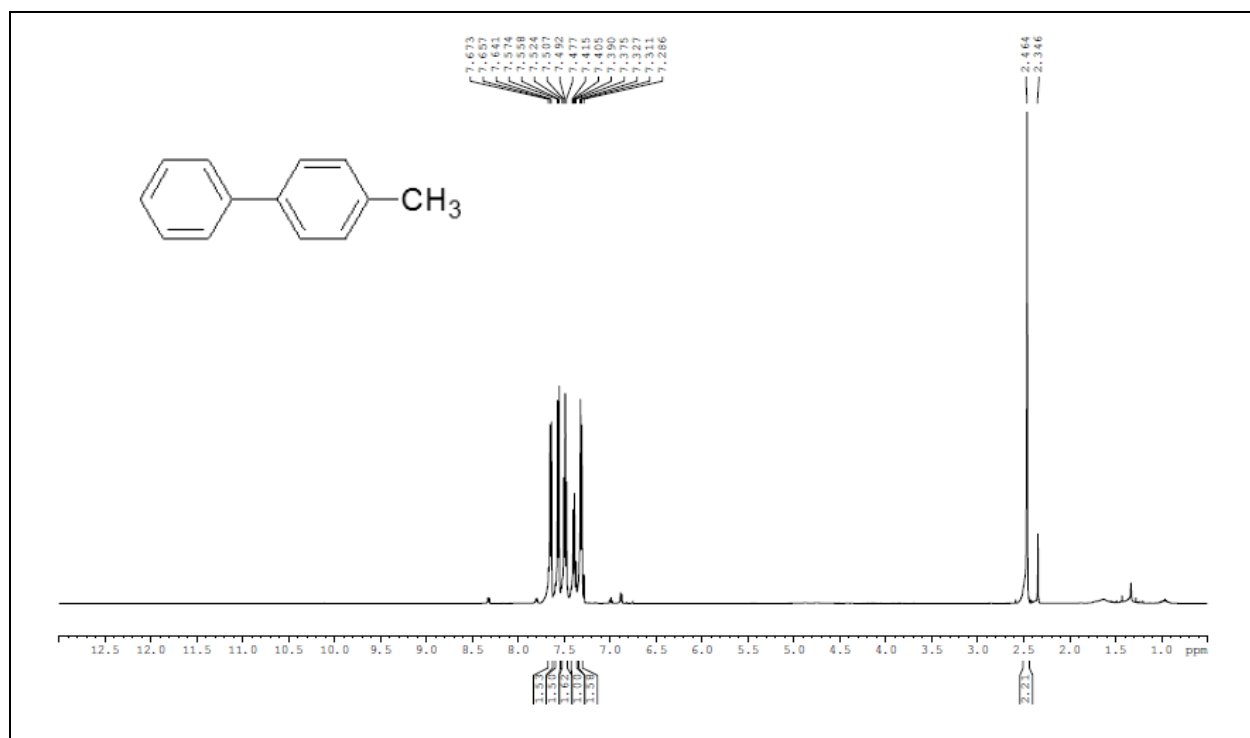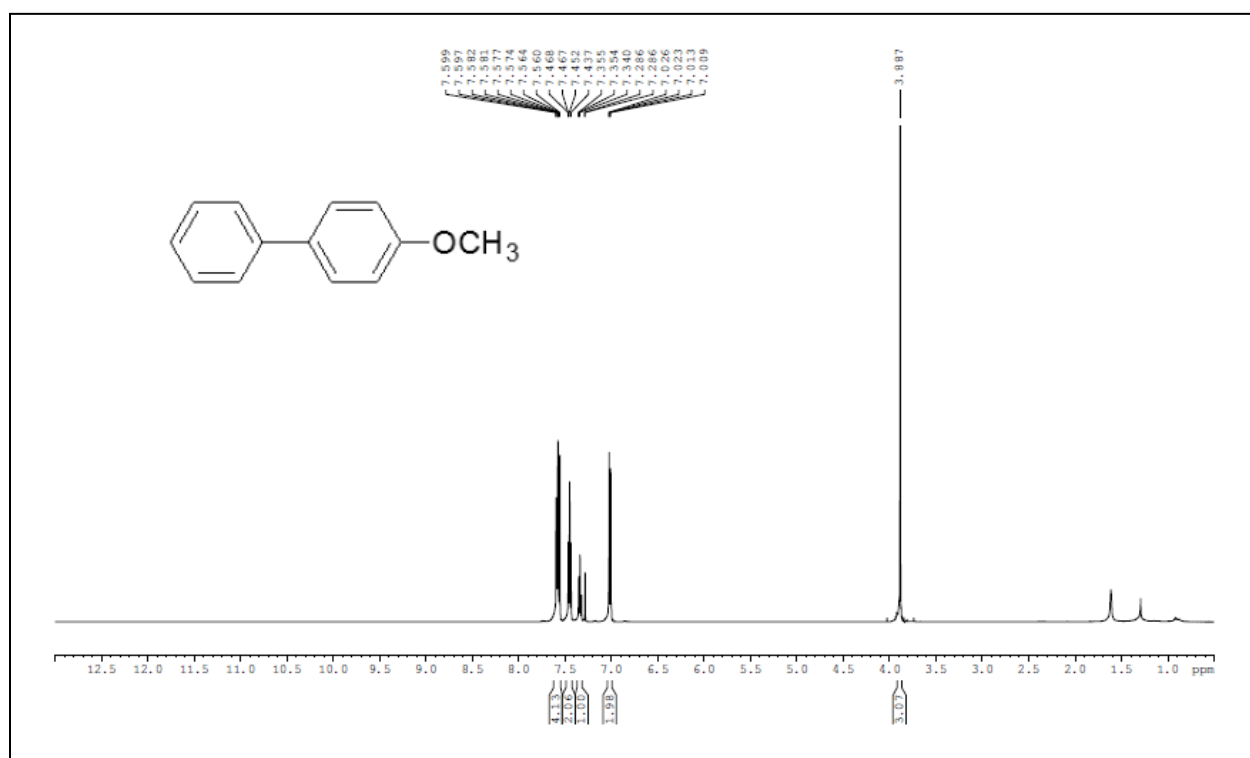

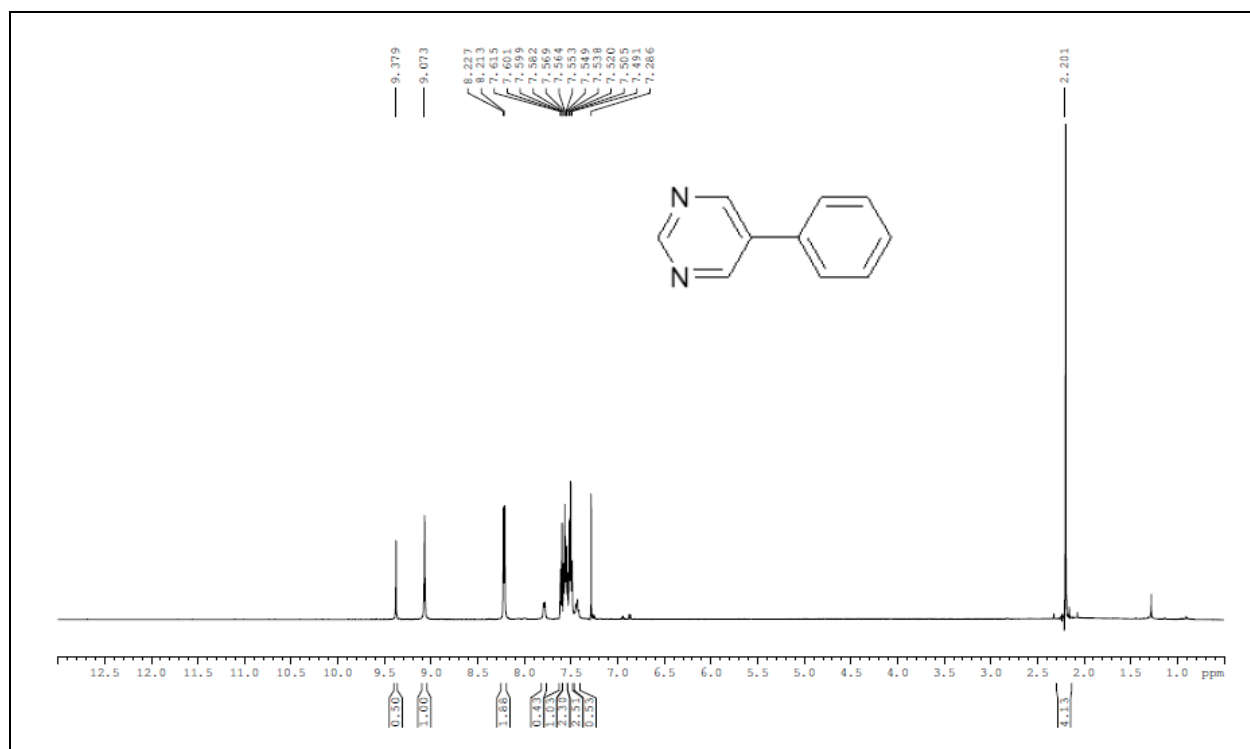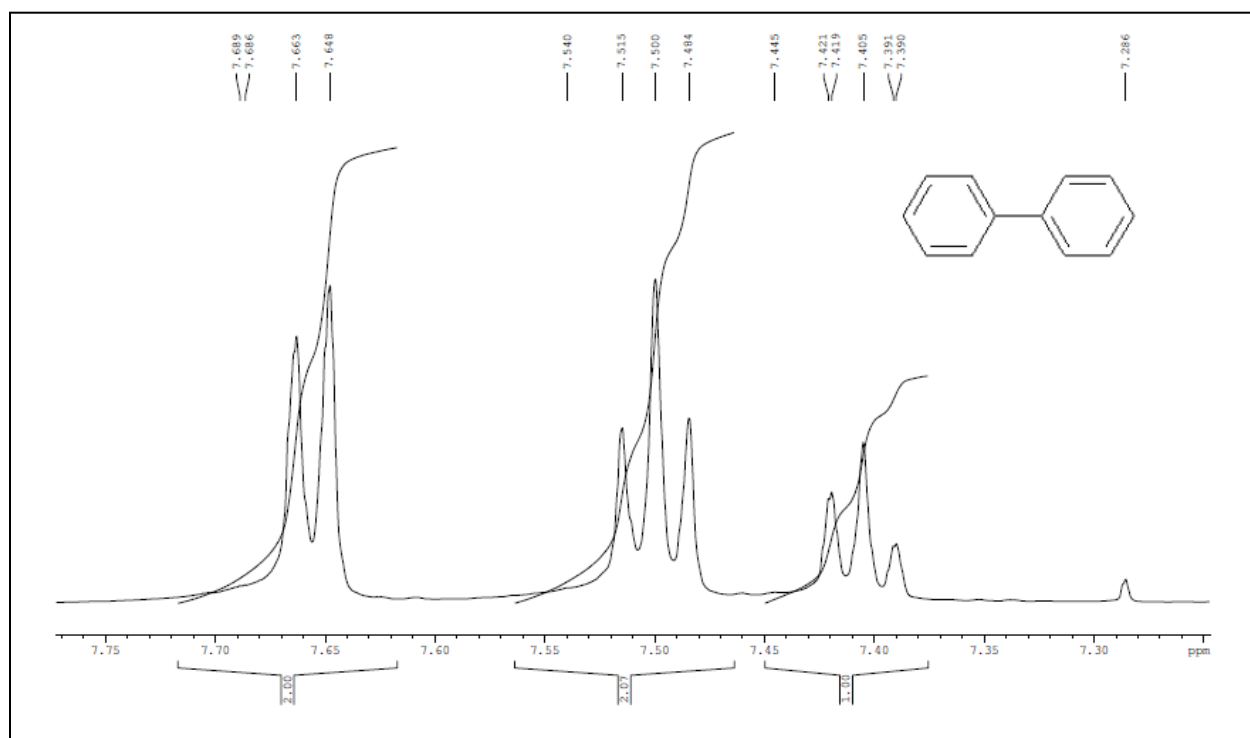

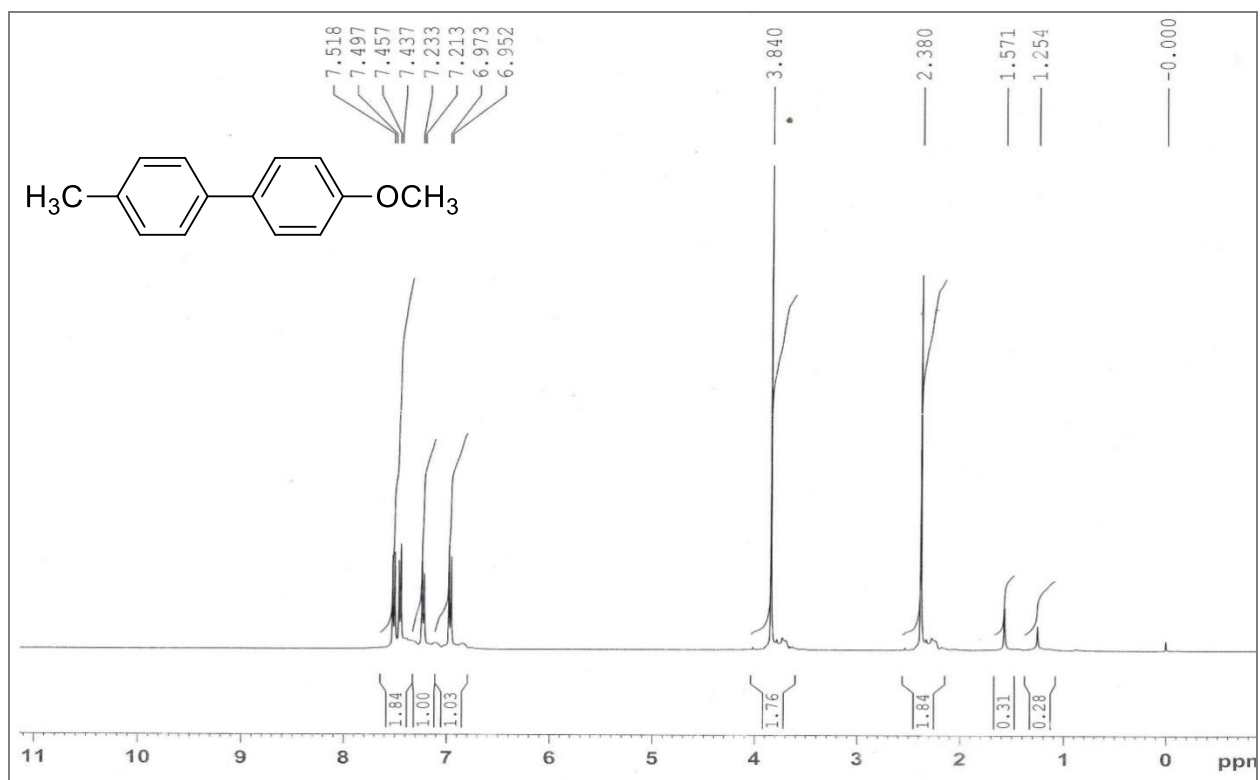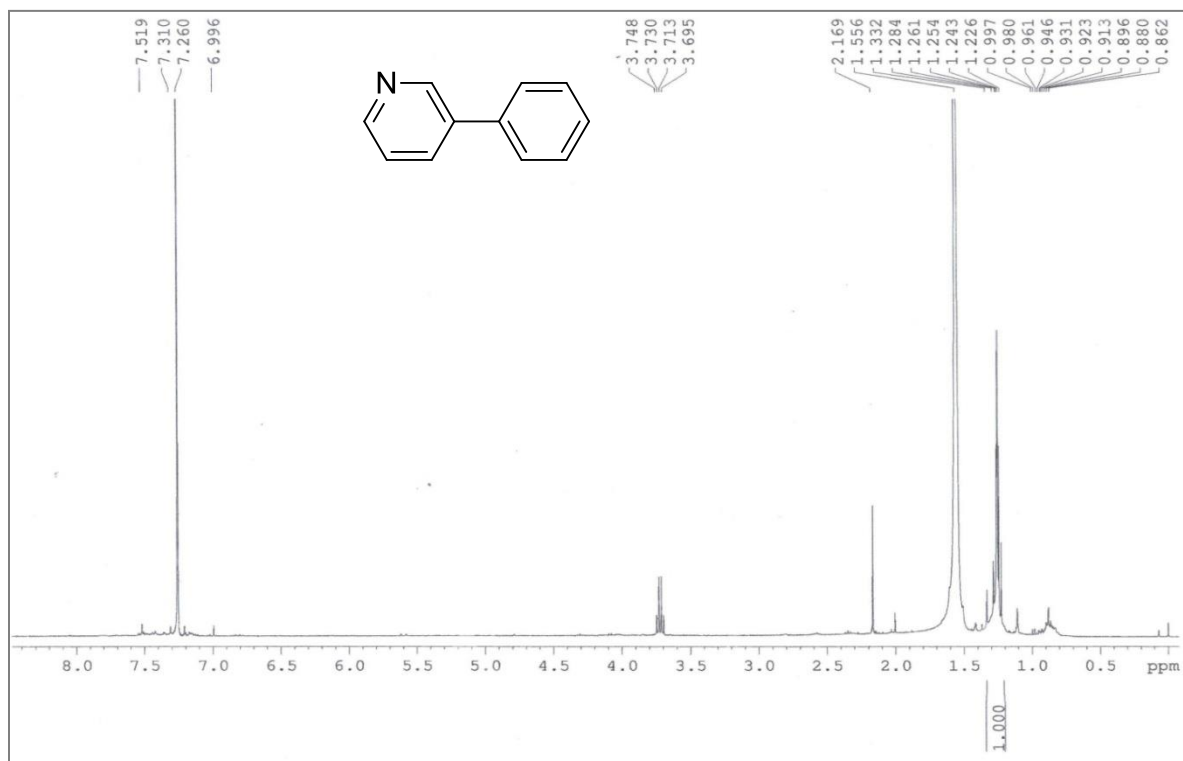

Supplement: S10 Fig — (PDF) [file pone.0184936.s010.pdf]

**S11 Fig.** Mass spectra of selected products for the Suzuki-Miyaura reaction.

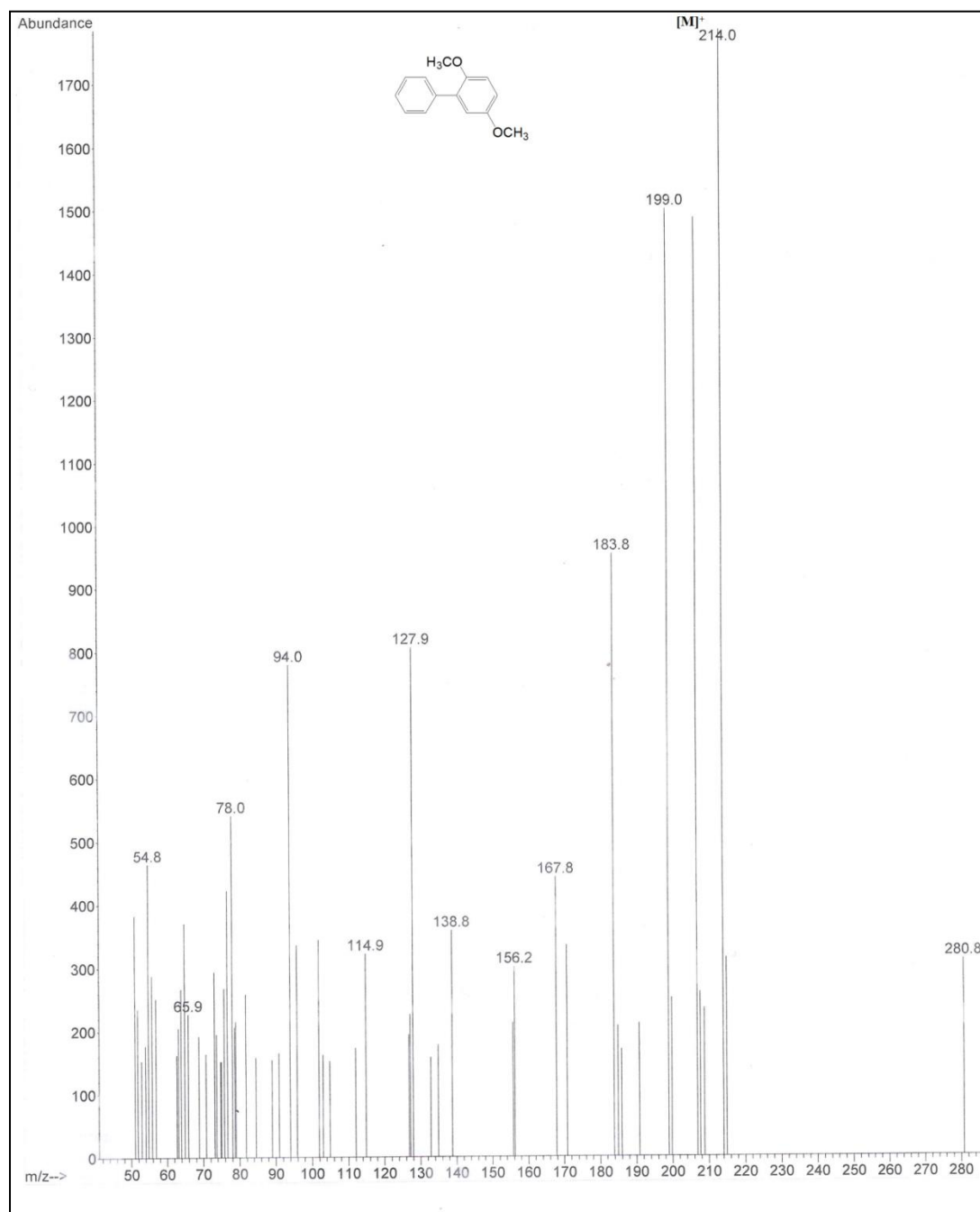

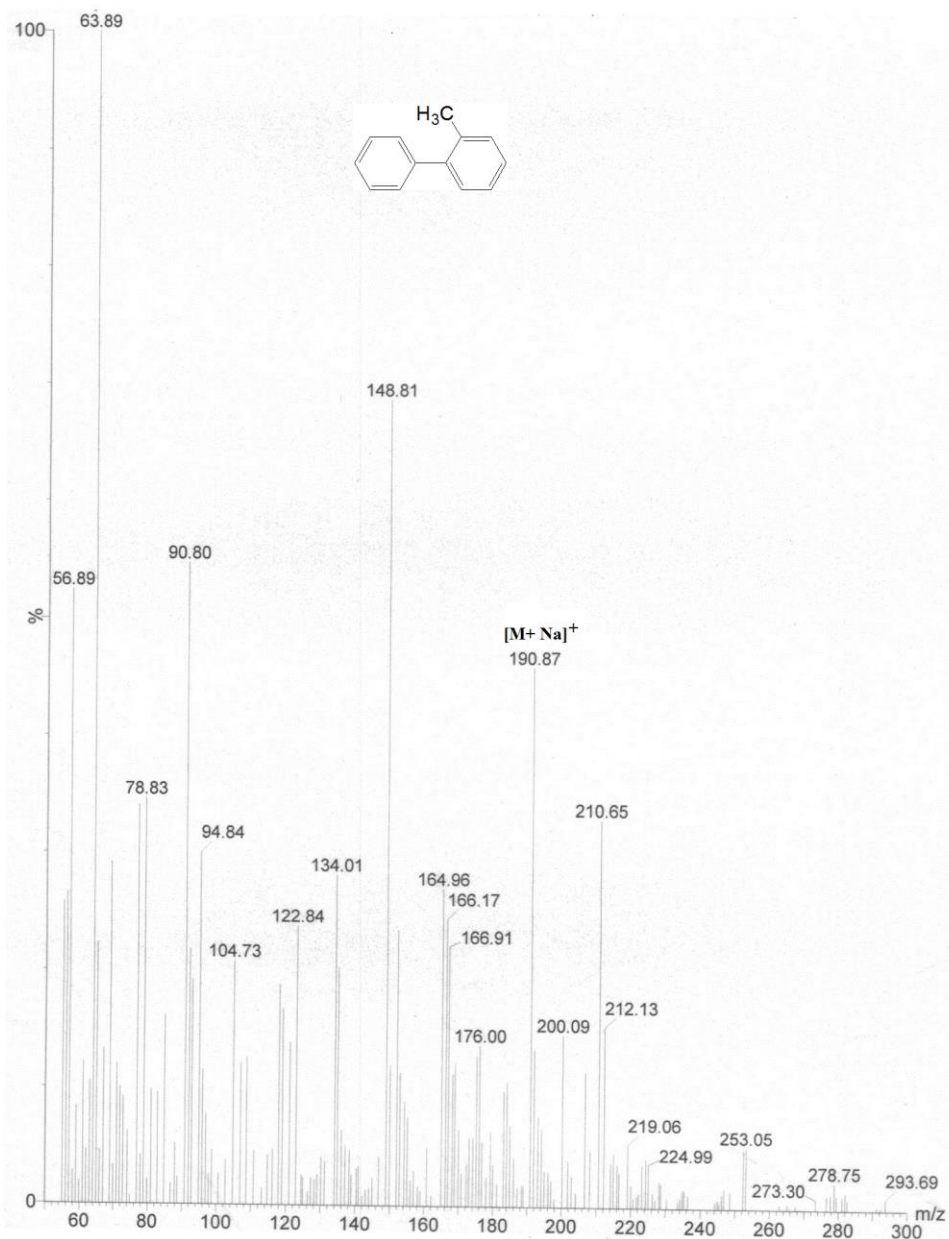

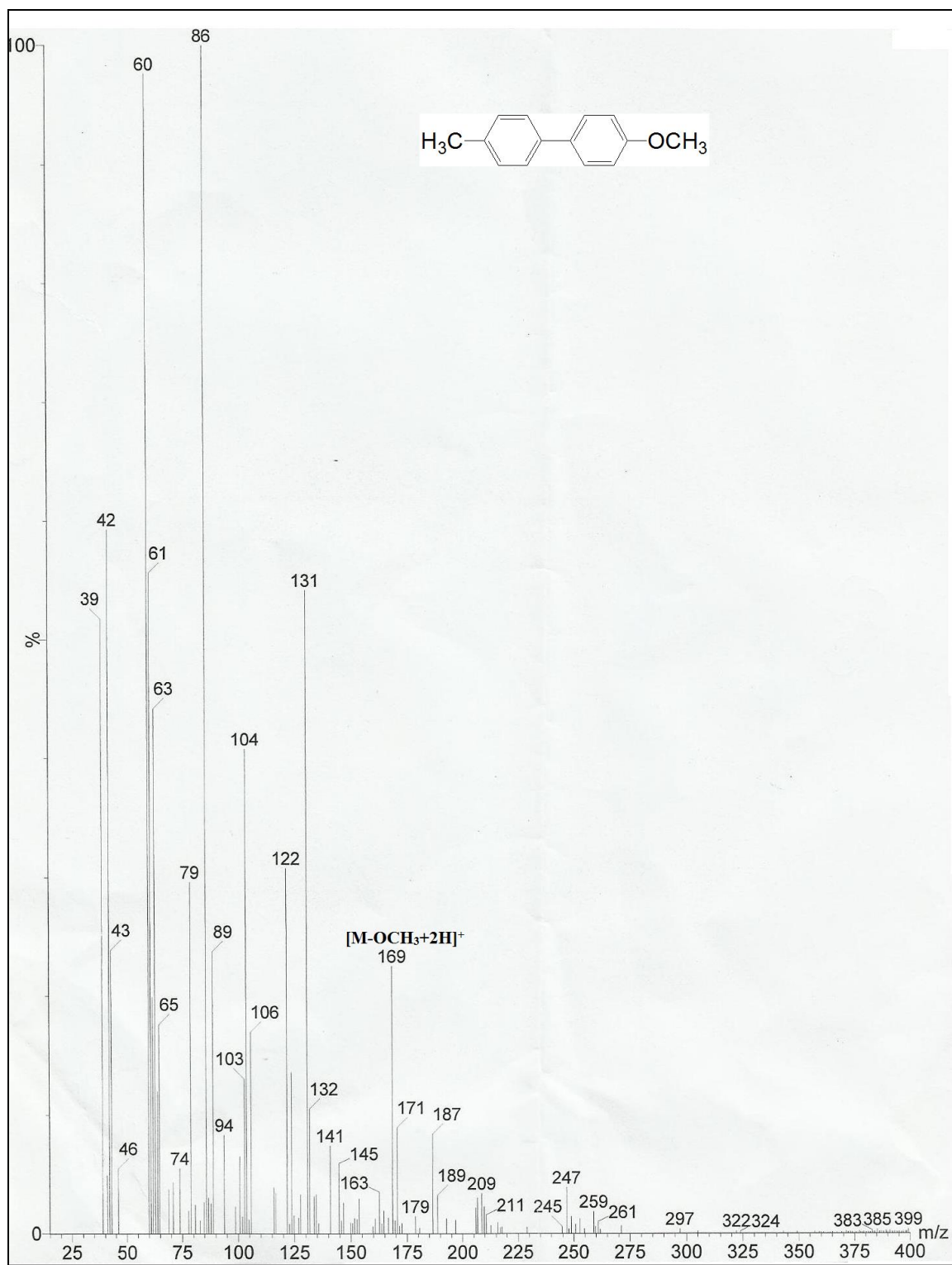

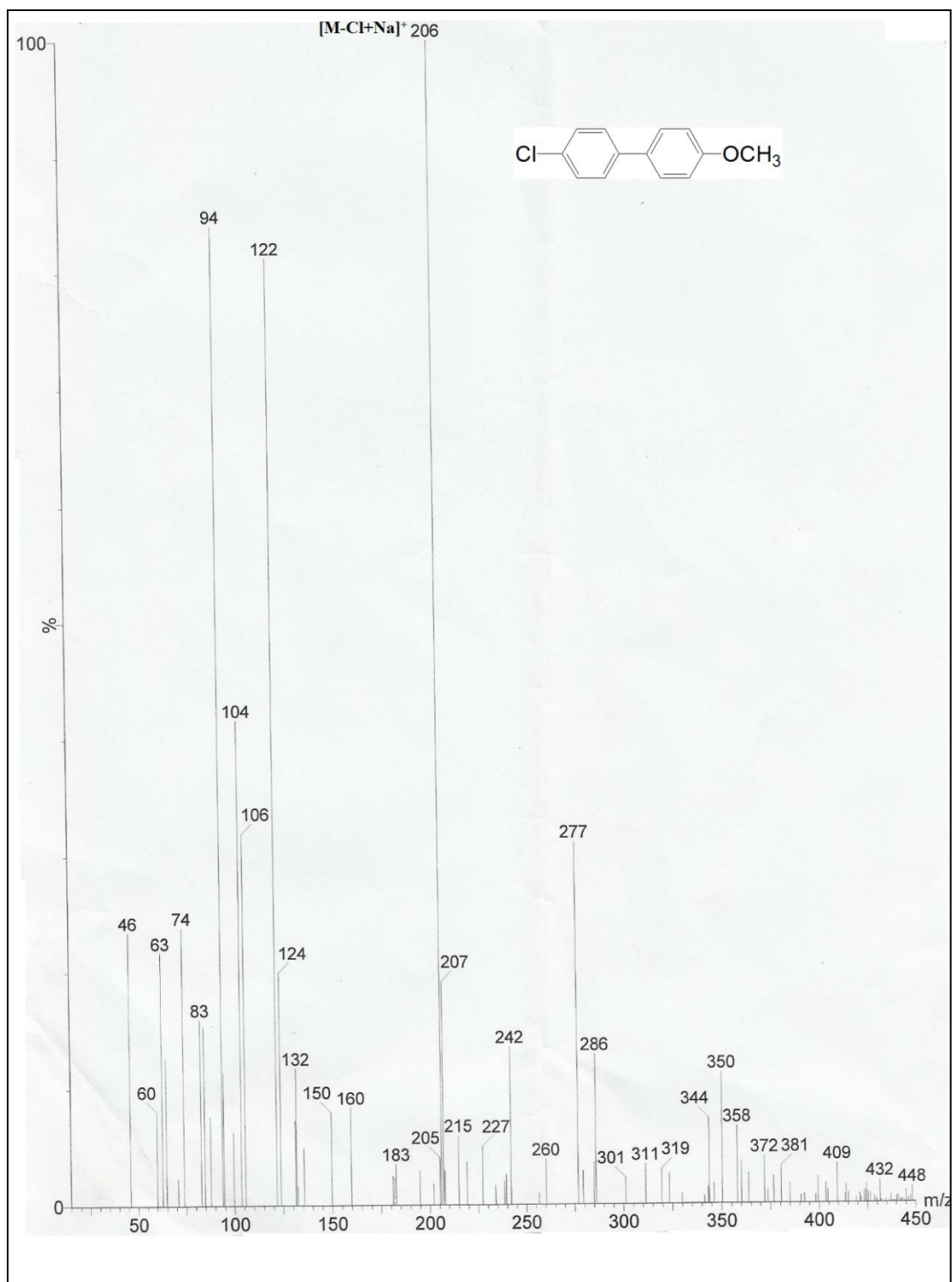

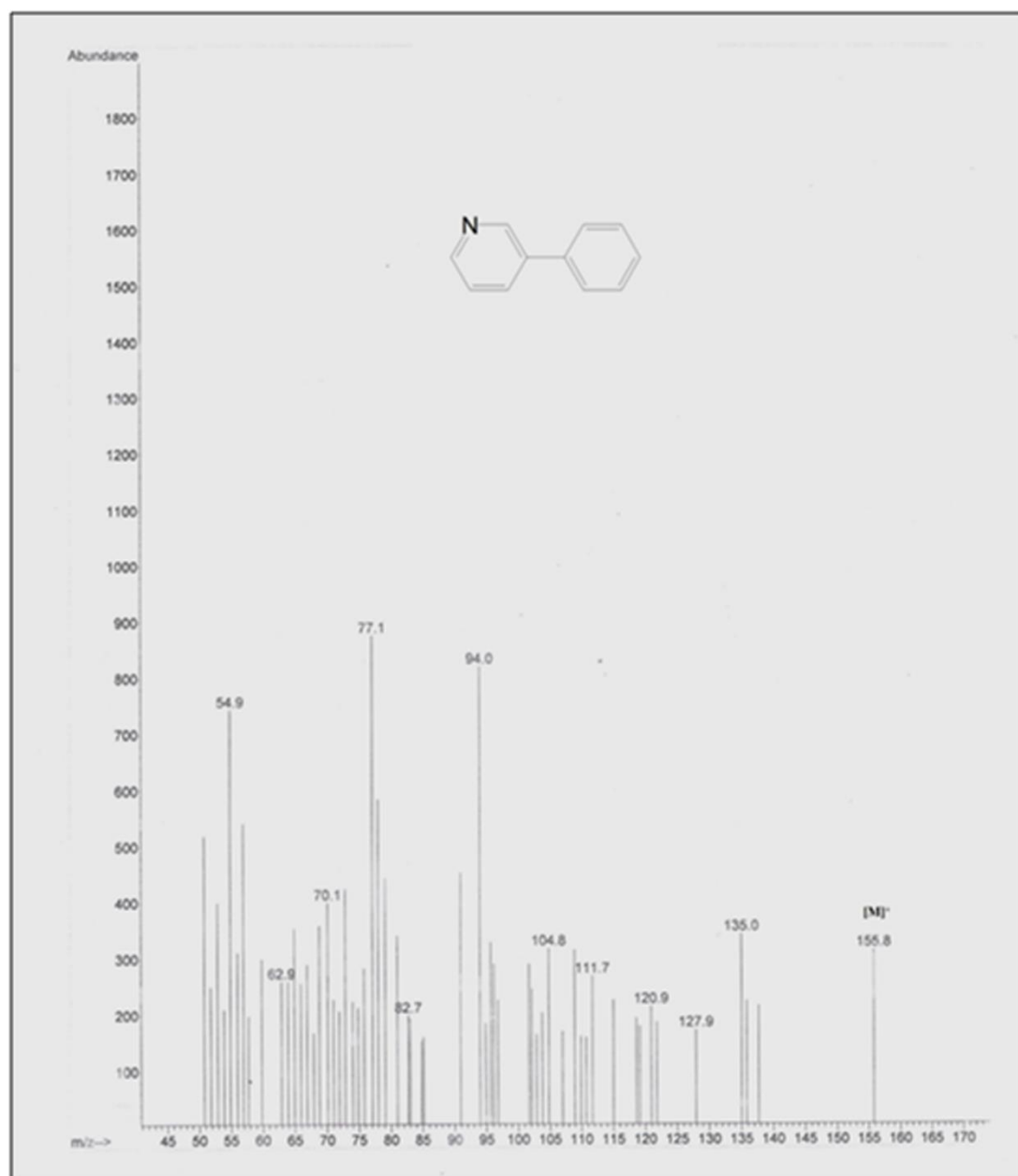

Supplement: S11 Fig — (PDF) [file pone.0184936.s011.pdf]

**S12 Fig.** Mass spectra of products for the alcohol-oxidation reactions

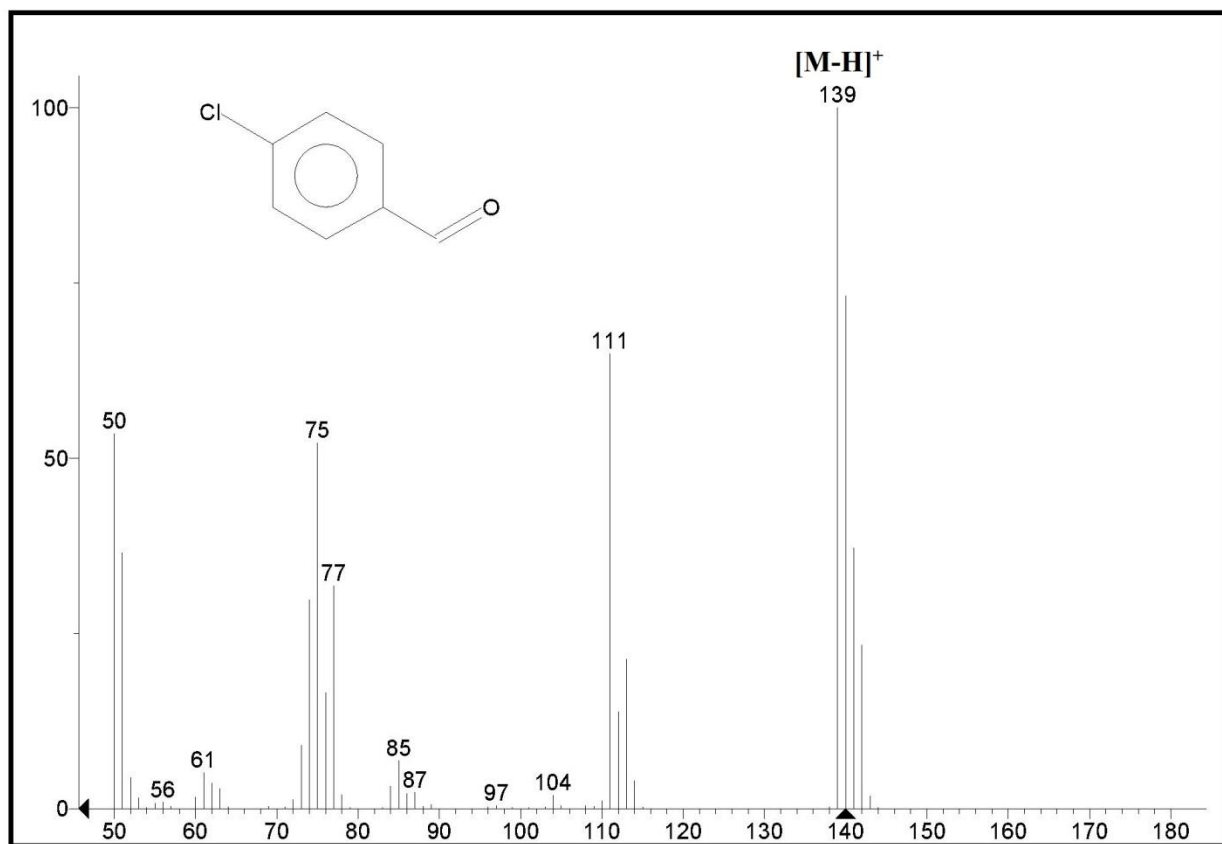

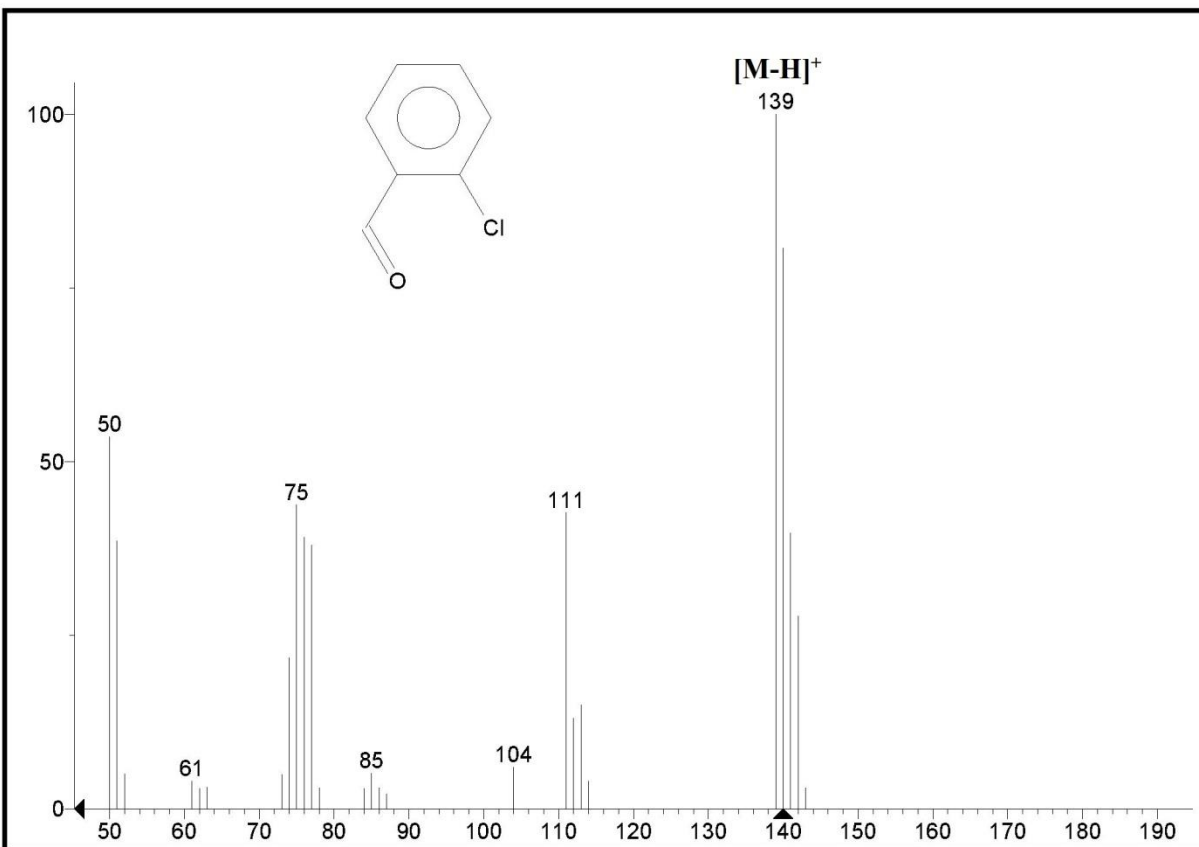

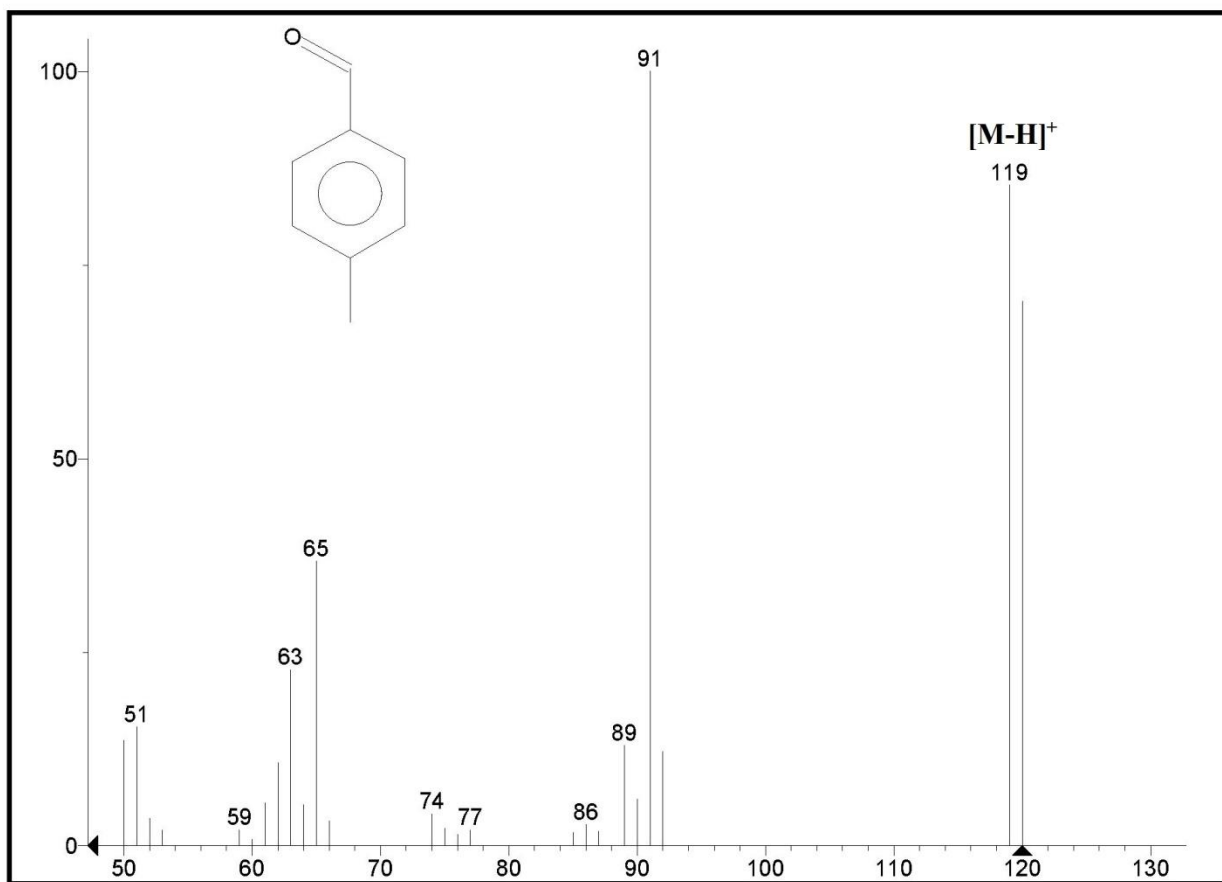

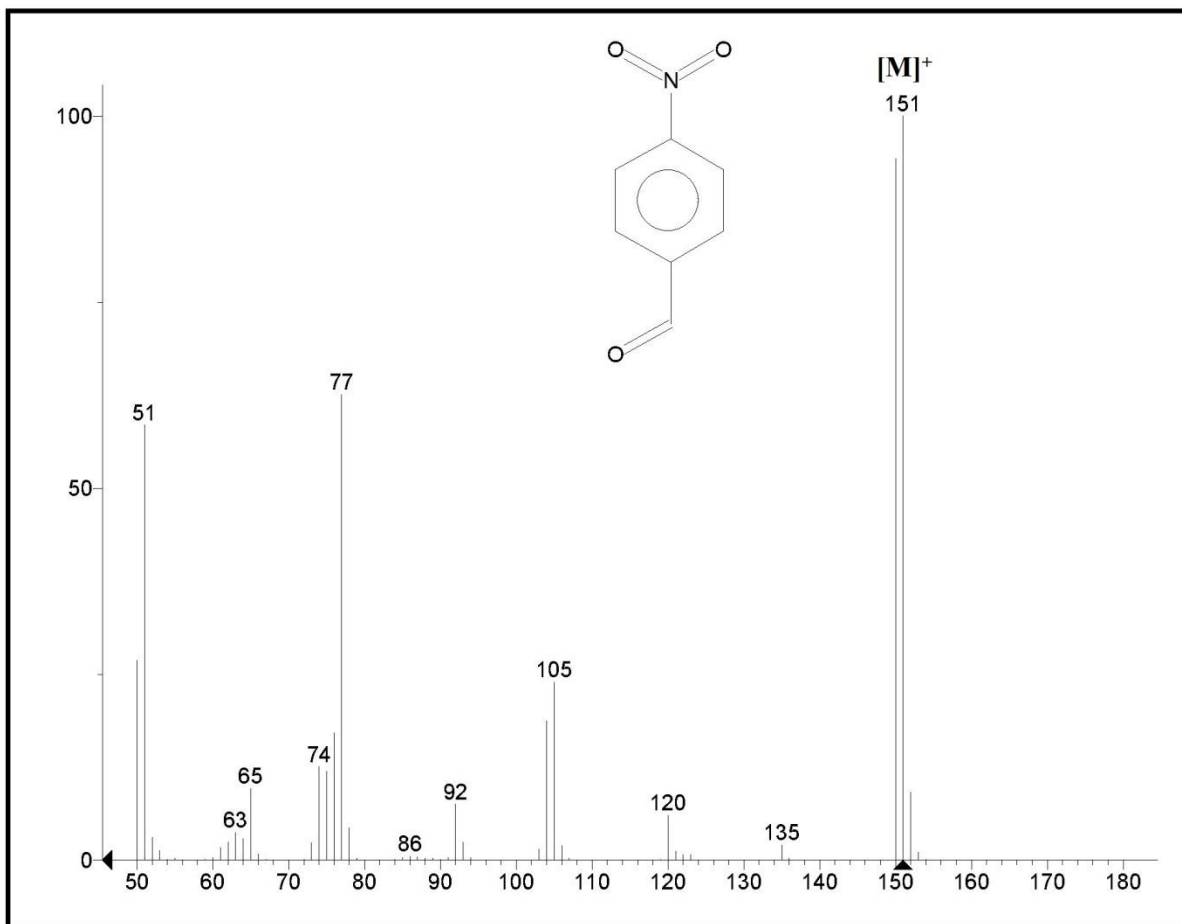

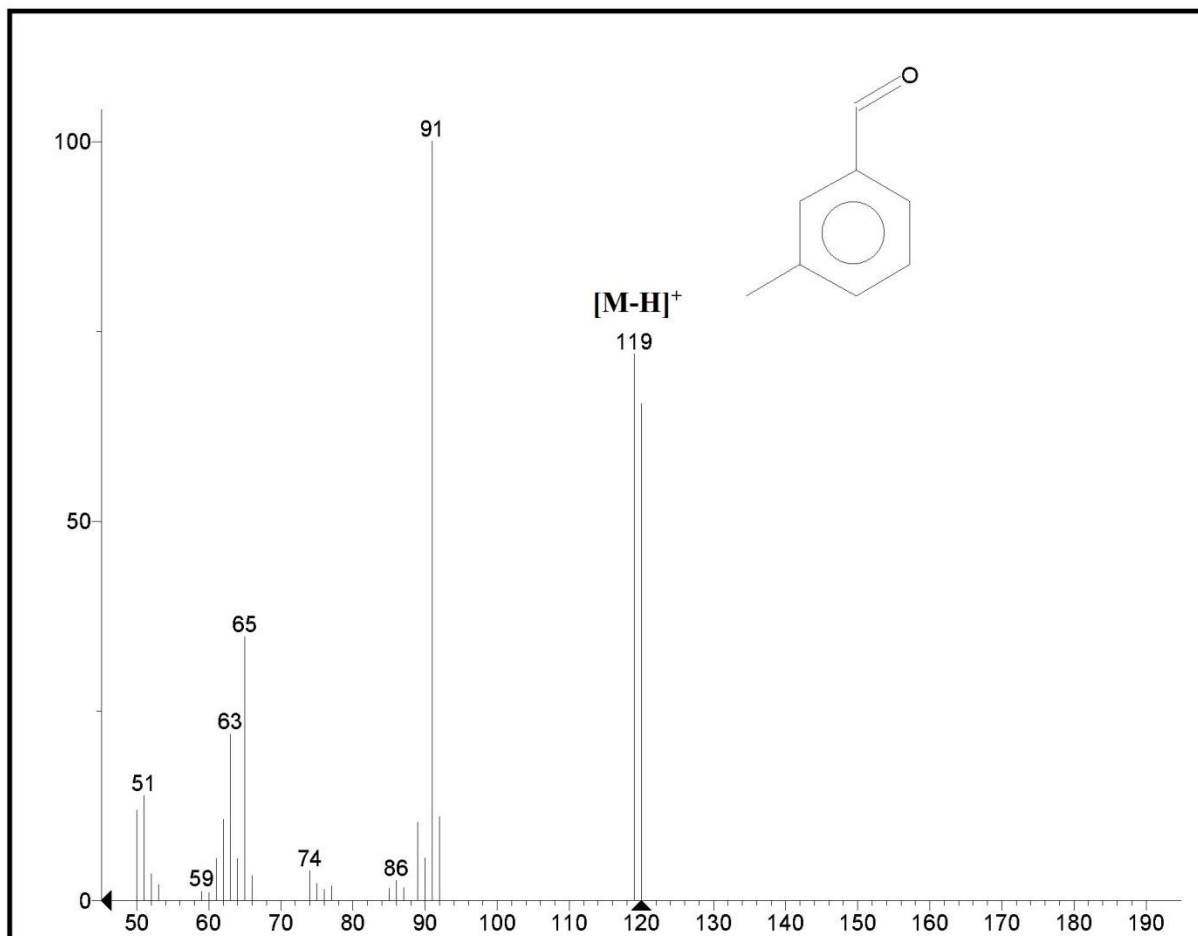

Supplement: S12 Fig — (PDF) [file pone.0184936.s012.pdf]
